# Postnatal enteral plasma supplementation following birth asphyxia increases fluid retention and kidney health in newborn pigs

**DOI:** 10.14814/phy2.70238

**Published:** 2025-02-05

**Authors:** Jingren Zhong, Stanislava Pankratova, Richard Doughty, Christoffer Kirkelund Flyger, Per Torp Sangild, Kerstin Skovgaard, Henrik Elvang Jensen, Duc Ninh Nguyen, Thomas Thymann

**Affiliations:** ^1^ Section for Comparative Pediatrics and Nutrition, Department of Veterinary and Animal Sciences University of Copenhagen Frederiksberg Denmark; ^2^ Department of Pathology Akershus University Hospital Lørenskog Norway; ^3^ Section for Pathobiological Sciences, Department of Veterinary and Animal Sciences University of Copenhagen Frederiksberg Denmark; ^4^ Department of Pediatrics Odense University Hospital Odense Denmark; ^5^ Department of Neonatology Rigshospitalet Copenhagen Denmark; ^6^ Department of Biotechnology and Biomedicine Technical University of Denmark Lyngby Denmark

**Keywords:** asphyxia, electrolytes, fluid, kidneys, neonates, plasma

## Abstract

Birth asphyxia can result in kidney dysfunction, disturbances in systemic electrolytes and fluid balance in newborns. Currently, there is no proven dietary approach to support asphyxiated newborns. This study investigates whether oral plasma supplementation improves kidney function and overall health in asphyxiated newborns. Cesarean‐delivered near‐term pigs with or without an 8 min intrauterine clamping of the umbilical cord were fed a milk replacer dissolved in water for 24 h in Experiment 1. Pigs were fed 72 h with milk replacers dissolved in either maternal plasma or water in Experiment 2. Blood, urine, and kidney tissue were collected for further analyses. Asphyxia disrupted blood electrolyte balance. And plasma feeding led to higher fluid retention for both asphyxiated and control pigs. Additionally, plasma feeding may also affect kidney development and protect kidneys from asphyxia induced impairments. Birth asphyxia in pigs led to immediate disturbance of electrolyte balance, impaired fluid retention, and kidney impairments. Plasma feeding may improve postnatal newborn hydration and may also improve the condition of kidneys following asphyxia.

## 1 INTRODUCTION

Neonatal asphyxia is a condition in newborns characterized by impaired placental gas exchange and nutritional delivery. It remains one of the most critical causes of neonatal morbidity and mortality (Lawn et al., 2005; Moshiro et al., 2019). The consequences of asphyxia are a combination of hypoxia, hypercapnia, acidosis, and hypotension (Antonucci et al., 2014; Durkan & Alexander, 2011). The kidneys are particularly sensitive to hypoxia due to their high metabolic rate and oxygen demand, and the portal vascular architecture (Bonnitcha et al., 2018; Gerosa et al., 2015; Shu et al., 2019). Asphyxia also results in the redistribution of cardiac output that reduces blood flow to the kidneys (Behrman et al., 1970; Cargill et al., 2020; Cohn et al., 1974). Besides, it predisposes the kidneys to oxidative stress (Perrone et al., 2007) and potentially triggers an inflammatory response (Okazaki et al., 2023). Consequently, asphyxia is likely the most prevalent cause of neonatal acute kidney injury (AKI) (Agras et al., 2004; Gallo et al., 2021). During the perinatal period, the kidneys play a crucial role in sensing and responding to systemic hypoxic stress (Lacombe et al., 1988) and regulating electrolytes and fluid homeostasis (Siegel, 1982). Kidney impairment following asphyxia may further influence postnatal body fluid and electrolyte balance with potential negative effects on blood pressure and tissue perfusion.

Following birth asphyxia, acute resuscitation typically involves positive ventilation, hypothermia, and medical treatment (Moshiro et al., 2019; Okazaki et al., 2023; Wagner et al., 1999). While less intense resuscitation strategies can be applied for animals with perinatal asphyxia, therapeutic approaches include stimulants for respiration and hormone secretion, as well as supplements for antioxidation (Sanchez‐Salcedo et al., 2019). After birth asphyxia, neonatal infants and animals require early enteral feeding to establish a normal growth trajectory and reduce adverse post‐asphyxia sequelae (Alonso‐Spilsbury et al., 2005; Jhajra et al., 2024). It is well known that breastfeeding is crucial for optimal postnatal growth and development (M'Rabet et al., 2008; Stuebe, 2009; Victora et al., 2016; Walker, 2004; Weström et al., 2020). However, neonatal asphyxiated infants or animals may have difficulties in obtaining adequate breastfeeding primarily due to neurological damage (Alonso‐Spilsbury et al., 2005; Kritzinger et al., 2017; Krüger et al., 2019). Asphyxiated neonates would therefore be dependent on nutritional supplementation via milk replacers. However, there is a paucity of information regarding the effects of milk replacers on cell‐ and organ‐repair in both asphyxiated human and animal neonates.

Specifically, for human and porcine neonates, it is not feasible to collect colostrum in adequate amounts from other mothers or sows. Colostrum and milk partly consist of components synthesized by the alveolar cells and partly derivatives from the maternal blood plasma (Hassiotou & Geddes, 2013; Truchet & Ollivier‐Bousquet, 2009). Plasma contains numerous bioactive substances such as immunoglobulins, coagulation proteins, complement proteins, regulatory peptides, microRNA, and key electrolytes that may be beneficial for development and stress responses. Given its valuable bioactive components and convenient availability, plasma has the potential to be an effective additive to milk formulas for both infants and animals. Plasma has been widely used in weaning pigs to improve growth and health (Balan et al., 2021). However, whether plasma can benefit newborns with birth complications remains unexplored. In this study, we hypothesized that plasma provides compounds with reparative effects on newborns subjected to birth asphyxia.

In the present study, we developed a new pig model to mimic the conditions of intrauterine asphyxia experienced by human and pig fetuses. From samples of brain, liver, and gut from the same experimental animals, we have previously demonstrated that moderate birth asphyxia induces brain and gut damage, and that plasma feeding supports organ repair after asphyxia (Nordsten et al., 2024; Ventura et al., 2024). The aim of this study was to examine the effects of asphyxia and oral plasma feeding on body fluid balance, electrolyte levels, and kidney health.

## 2 MATERIALS AND METHODS

### 2.1 Experimental model

**FIGURE 1:**
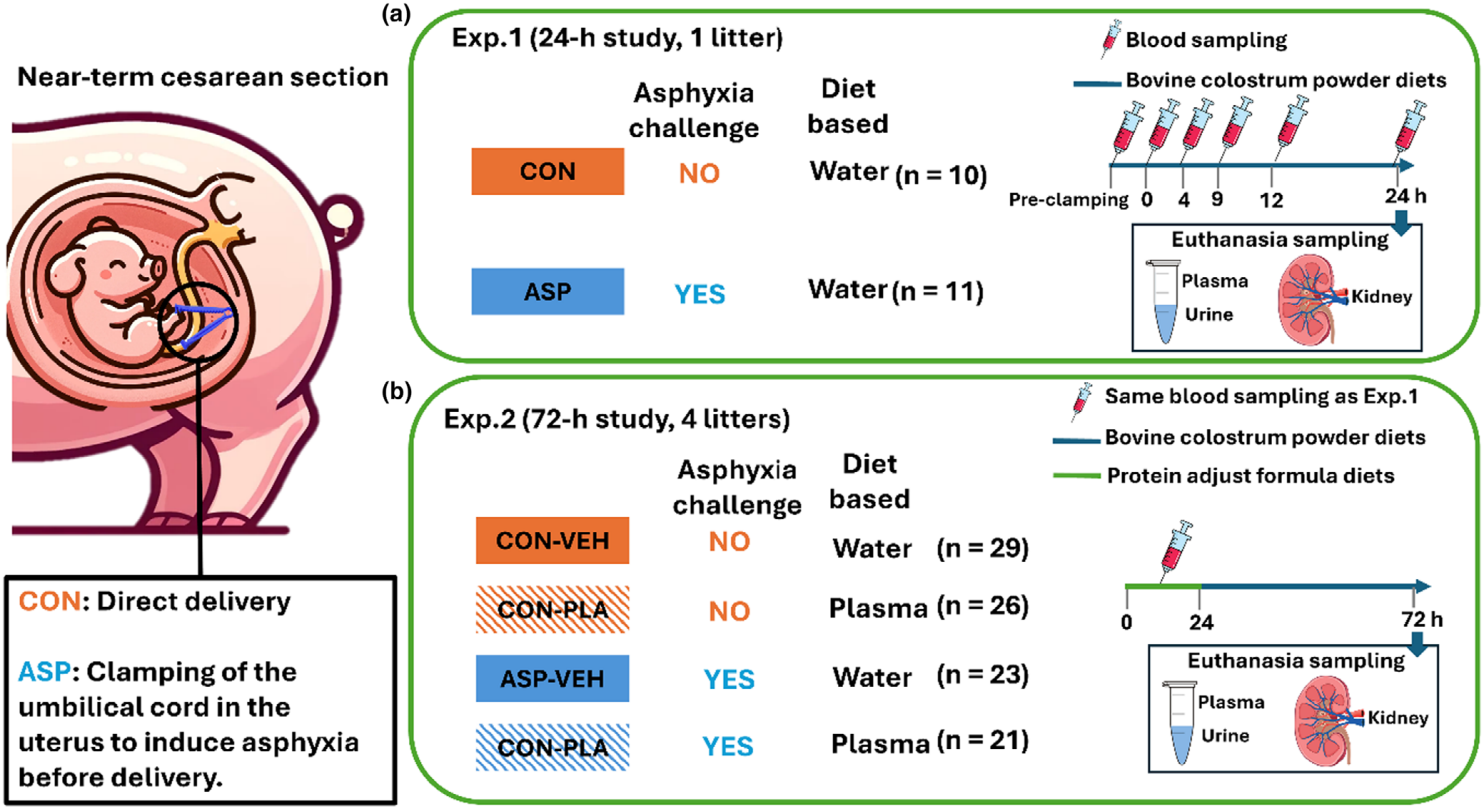
Study design. (a) Experimental design of the 24‐h study: Newborn near‐term piglets gained from 1 L (*n* = 21) were either directly delivered or subjected to an asphyxia condition (CON/ASP). The pigs were fed with a colostrum diet prepared on water. Pigs were euthanized at 24 h. (b) Experimental design of the 72‐h study: Newborn near‐term piglets gained from 4 L (*n* = 99) were either directly delivered (CON) or subjected to an asphyxia condition (ASP). For the initial 24 h, the pigs were fed with a protein‐adjusted colostrum diet prepared on sow plasma or water. From 24 h to 72 h, the diets of pigs were switched to a protein‐adjusted formula diet prepared on either sow plasma or water (PLA/VEH). Pigs were euthanized at 72 h.

The animal experiment protocol (license number, 2020‐15‐0201‐00520) was approved by the Danish Animal Experiments Inspectorate prior to conducting the experiment. The study design is shown in Figure 1. The experiment involved delivering near‐term piglets (gestational age 113 days, term = 116 ± 2 days) from five sows (Large White × Danish Landrace × Duroc) through cesarean section as previously described (Sangild et al., 2013). During the surgery, piglets were either delivered without prior cord clamping (Control, CON) or subjected to 8 min cord clamping (Asphyxia, ASP). To verify induction of asphyxia we collected arterial cord blood before and after clamping for blood gas measurements. Immediately after delivery, all pigs were given an intramuscular injection of 0.1 mL of doxapram (Dopram, 20 mg/mL, Carinopharm GmbH, Eime, Germany; #12360318) and 0.1 mL of flumazenil (0.1 mg/mL, Hameln pharma GmbH, Hameln, Germany; #036259) to stimulate breathing activity and counteract the effects of the zolazepam given to mother for sedation, respectively. Intensive resuscitation was then applied to asphyxiated pigs in a manner similar to that used for asphyxiated infants, aiming to stabilize pulmonary and circulatory function as quickly as possible. This included immediate airway clearance, stimulation to encourage breathing, and positive pressure ventilation as needed. Following stabilization, the pigs were reared in individual incubators and received parenteral nutrition support via an implanted umbilical catheter, as previously described (Sangild et al., 2013). After C‐section, maternal sow's blood was collected aseptically into heparinized glass bottles using a vacuum system. The blood was centrifuged and plasma was isolated and stored until use.

To assess the effects of asphyxia over the first 24 h after birth (Exp. 1, 24‐h study), pigs from 1 L (Figure 1a), that is, Control (CON, *n* = 10; 5 female +5 male) and Asphyxia (ASP, *n* = 11; 6 female +5 male), were fed with a diet prepared by dissolving bovine colostrum powder (100 g/L, Biofiber‐Damino, Gesten, Denmark; #5500102014) and bovine whey protein powder (70 g/L; Lacprodan® DI‐9224, Arla Food Ingredients, Denmark) and vitamins/minerals (2 g/L; Phlexy Vits, Nutricia, UK; #80173) in water. In Experiment 2, control and asphyxiated pigs from four litters were reared for 72 h (referred to as Exp. 2, 72‐h study). For the initial 24 h, the pigs were fed with either bovine colostrum powder mixed with maternal sow's plasma (PLA) or bovine colostrum powder mixed with water supplemented with bovine whey protein powder (70 g/L; Lacprodan® DI‐9224, Arla Food Ingredients, Denmark) to ensure equal protein content between the groups (vehicle, VEH). From 24 h to 72 h, we replaced bovine colostrum powder with bovine dairy powders that were also dissolved in either sow's plasma or water. Details for diet composition and nutritional content are shown in Table S1. Therefore, the 72‐h study employed a two‐by‐two factorial design, that is, with or without asphyxia (ASP/CON) and with or without plasma (PLA/VEH). Consequently, the four experimental groups were as follows: CON‐VEH (*n* = 29; 14 female +15 male), CON‐PLA (*n* = 26; 14 female +12 male), ASP‐VEH (*n* = 23; 11 female +12 male), and ASP‐PLA (*n* = 21; 10 female +11 male). We established permanent umbilical and esophageal catheters in both Exp 1 and Exp 2 as outlined previously (Sangild et al., 2013), and provided the first oral feeding 5 h after birth, followed by feeding every 3 h. Following 72 h rearing in Exp.2, all pigs from the 4 L were tissue collected as specified below (total *n* = 99, see Figure 1b).

### 2.2 Tissue collection

Following anesthesia, blood was collected via heart puncture sampling into a heparin vacutainer (BD Diagnostics, Oxford, UK). Subsequently, the pigs were euthanized through intracardiac injection of sodium pentobarbital (60 mg/kg). Kidney cortex tissue, urine, and plasma were collected immediately. The kidneys were cut longitudinally to include the renal capsule, cortex, medulla, and pelvis. Half of the tissue was stored in a histological cassette and fixed in 4% phosphate‐buffered paraformaldehyde, while the other half was snap‐frozen in liquid nitrogen and stored at −80°C for further analysis. Urine was obtained through cystocentesis, and plasma was collected after centrifugation of blood at 2500 × *g* for 10 min at 4°C. Both urine and plasma were immediately stored at −20°C.

### 2.3 Tissue histology

The fixed kidney tissues were dehydrated through a series of graded ethanol and xylene solutions, then embedded in paraffin. The paraffin blocks were sectioned at 4 μm and stained with hematoxylin and eosin (H&E). The renal histopathological manifestations were evaluated according to a 2‐point scale (none; present) by two independent evaluators without prior knowledge of the treatment groups. The prevalence of renal histopathological changes was calculated as the ratio of samples with histopathological changes to the total number of samples. All histology quantification analyses were performed using ImageJ software version 1.50i (NIH, Bethesda, MD).

### 2.4 Blood gas and biochemical analysis

Blood gas measurements were performed using GEM Premier 3000 (Instrumentation Laboratory, USA). The Advia 1800 Chemistry System, Siemens Healthcare, Ballerup, Denmark, was used to analyze the biochemistry of plasma and urine. The estimated glomerular filtration rate (eGFR) was calculated (Gasthuys et al., 2017).

### 2.5 Gene expression analysis

After defrosting, kidney total RNA was extracted using the RNeasy Mini Kit (Qiagen, Copenhagen, Denmark; #74106) following the manufacturer's protocol. The purity and concentration of RNA were measured with Nanodrop ND‐1000 (Saveen Werner AB, Limhamn, Sweden). RNA integrity was analyzed using an Agilent 2100 Bioanalyzer (Agilent Technologies, Nærum, Denmark) and the RNA 6000 Nano Kit (Agilent Technologies, Nærum, Denmark; #5067‐1512). cDNA was synthesized from 2000 ng of total RNA using the High‐Capacity cDNA Reverse Transcription Kit (Thermo Fisher Scientific, United States; #4368814). Gene expression was measured using 96.96 Dynamic Array Integrated Fluidic Circuits (Standard BioTools, South San Francisco, CA), as previously described (Skovgaard et al., 2013). The primers used are shown in Table S2. Relative amplicon concentrations and dynamic range of the assays were determined with standard curves from three separate dilution series of pooled cDNA. To control genomic DNA and unspecific amplifications, non‐reverse transcribed RNA and no‐template reactions were included. The data acquired in Fluidigm Real‐Time qPCR Analysis software 3.0.2 were processed with GenEx5 (MultiD, Gothenburg, Sweden). The reaction efficiency of each gene was used for data correction. After identifying the most optimal reference genes, four genes were used to normalize the gene expression data: Hypoxanthine Phosphoribosyltransferase 1 (*HPRT1*), ribosomal protein L13a (*RPL13A*), peptidylpropyl isomerase A (*PPIA*), and Tyrosine 3‐Monooxygenase/Tryptophan 5‐Monooxygenase Activation Protein Zeta (*YWHAZ*). The relative expression results were presented as fold changes to the CON‐VEH group in the 72‐h study.

### 2.6 Fluorescent RNAscope in situ hybridization

RNA in situ hybridization was performed using an RNAScope® Multiplex Fluorescent V2 Assay (Advanced Cell Diagnostics, Hayward, California, USA; #323110) with a HybEZ™ II Hybridization System (Advanced Cell Diagnostics, Hayward, California, USA; #321711) following the manufacturer's instructions. Paraffin‐embedded kidney tissues (*n* = 3 per group) were sectioned and mounted on Super Frost glass slides (Thermo Scientific; #10457673). The deparaffinized sections were washed in PBS and pretreated according to the manufacturer's instructions. Pig‐specific probes were used to detect succinate receptor and lactate receptor (RNAscope® Probe ss‐SUCNR1; #1153641 and ss‐HCAR1; #833471, respectively), while control probes (RNAscope® Probe ss‐PPIB; #428591; RNAscope® 3‐plex Negative Probe; #320871, all from Advanced Cell Diagnostics) were employed. Opal 520 (1:1000; Akoya Biosciences, MA, USA; #FP1487001KT) was used for visualization. Cell nuclei were counterstained with DAPI (Invitrogen, #D1306). Images were captured using a Zeiss Axioobserver microscope equipped with an Axiocam 702 camera.

### 2.7 Data analysis and statistics

The comparison of survival curves was done in GraphPad 10.4.1 using Gehan‐Breslow‐ Wilcoxon test. Other statistical analyses were performed using R Studio 4.2.0 (R Studio, Boston, MA, United States). Continuous data were fitted into a linear mixed‐effect model using the lme4 and multcomp packages. The model included treatment, sex, and birth weight as fixed factors, and litter as a random factor. Subsequent to the initial analysis, a post hoc comparison was conducted between groups except for CON‐VEH versus ASP‐PLA and CON‐PLA versus ASP‐VEH as these comparisons were not biologically relevant. *p* values for the main effects of plasma (PLA vs. VEH, reported as *P*_PLA_) and asphyxia (ASP vs. CON, reported as *P*_ASP_), and their interaction effects (*P*_INT_) were generated with pairwise post hoc *t*‐tests from a similar linear model. Group comparisons for each gene in the Fluidigm gene expression analyses were FDR corrected. The data were assessed for normal distribution, and if necessary, logarithmic transformation was performed to achieve normality. The comparison of the prevalence of histological lesions between groups was done by Fisher's exact test in R. A *p* value of less than 0.05 was considered statistically significant.

## 3 RESULTS

This study is part of a larger program where also endpoints related to the brain (Ventura et al., 2024) and the gut (Nordsten et al., 2024) were investigated in tissues derived from the same animals as in the current study. Specifically the study by Ventura et al. (2024) showed that asphyxia leads to impaired postnatal motor function, brain histopathological changes, delayed maturation of preoligodendrocytes, neuronal apoptosis, and transient alteration in gene expression related to oxidative stress, inflammation, and synaptic functions in specific brain regions. They also demonstrated that plasma supplementation improved pig motor skills, attenuated neuronal apoptosis, and affected gene expression related to neuroinflammation, neurotransmission, and antioxidants. Moreover, Nordsten et al. (2024) found that asphyxia led to decreased weight gain and survival, while plasma feeding, independent of asphyxia, was associated with reduced gut lesions, decreased gut permeability and inflammation, and improved gut maturation and function. Some of the baseline data (such as body weight, survival, and some blood parameters etc.) reported in the current manuscript, are also reported in the previous two papers (Nordsten et al., 2024; Ventura et al., 2024).

### 3.1 The effects of asphyxia on clinical and paraclinical parameters, blood gas, and renal histological lesions during first 24 h

**FIGURE 2:**
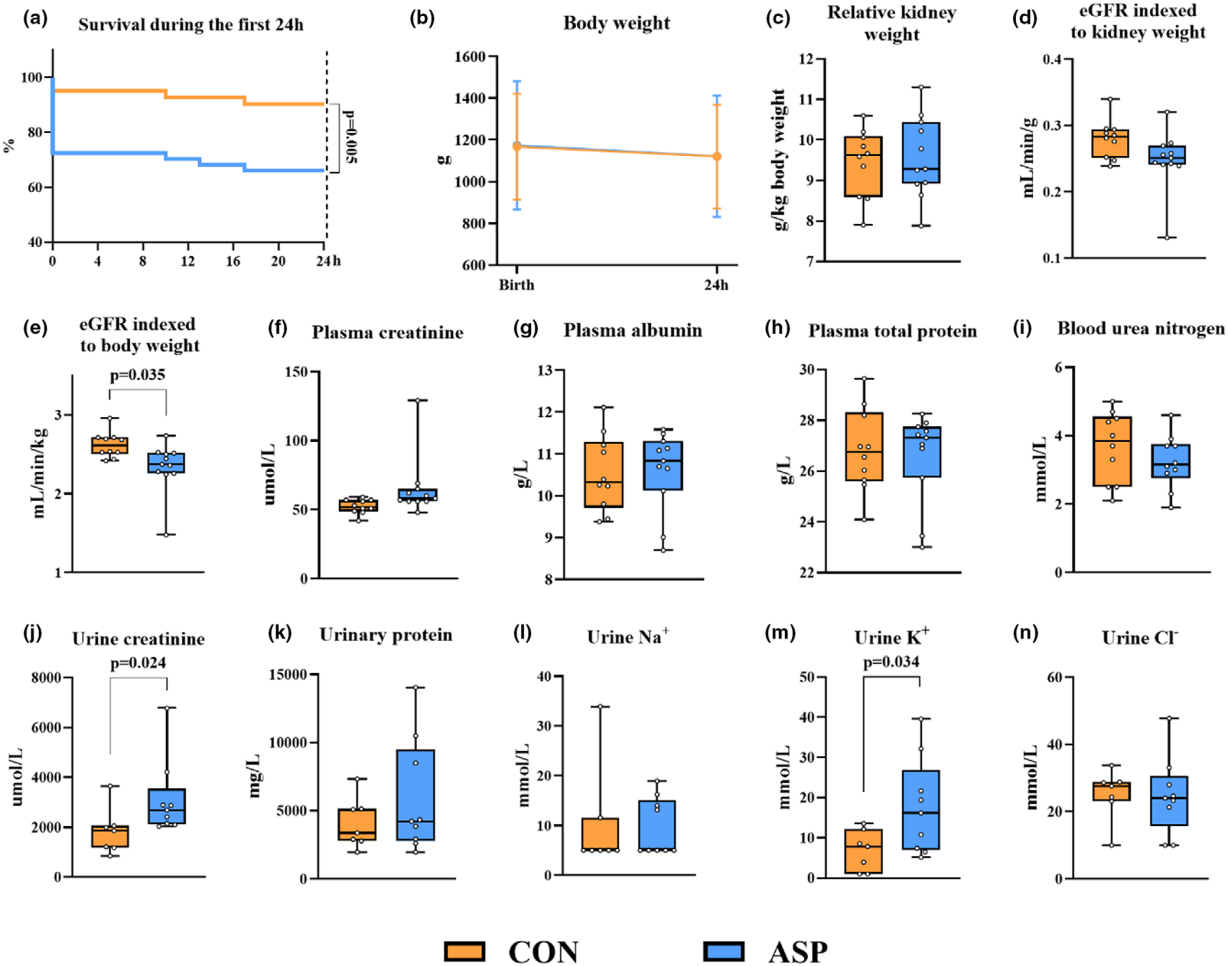
Effects of asphyxia on clinical outcomes, blood, and urine biochemistry in pigs during the first 24 h. (a and b) The survival rate and body weight of all vehicle‐fed pigs from all 5 L (*n* = 41 for control (CON), *n* = 47 for asphyxia (ASP)). (c) The relative kidney weight. (d and e) The estimated glomerular filtration rate (eGFR) indexed to kidney weight and body weight, respectively. (f–i) Plasma biochemistry. (j–n) Urine biochemistry. For panels c–n, *n* = 7–10 for CON and *n* = 8–11 for ASP.

**FIGURE 3:**
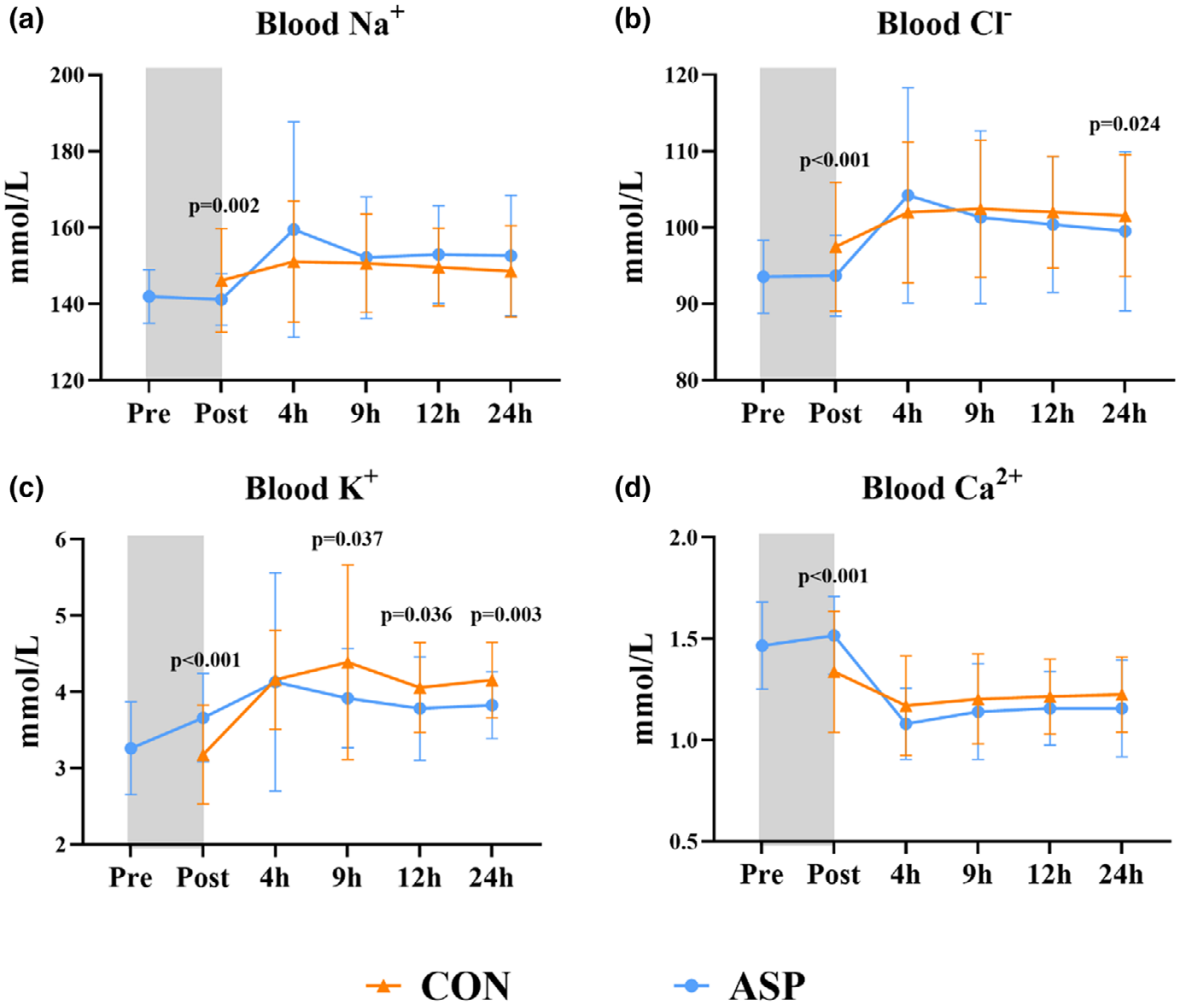
Effects of asphyxia on blood electrolyte levels in pigs during the first 24 h. (a–d) Blood Na^+^, Cl^−^, K^+^, and Ca^2+^ levels in vehicle‐fed pigs from all 5 L, respectively. All data for the control group (CON, *n* = 35) and asphyxia group (ASP, *n* = 27) are presented as the means ± SD.

**FIGURE 4:**
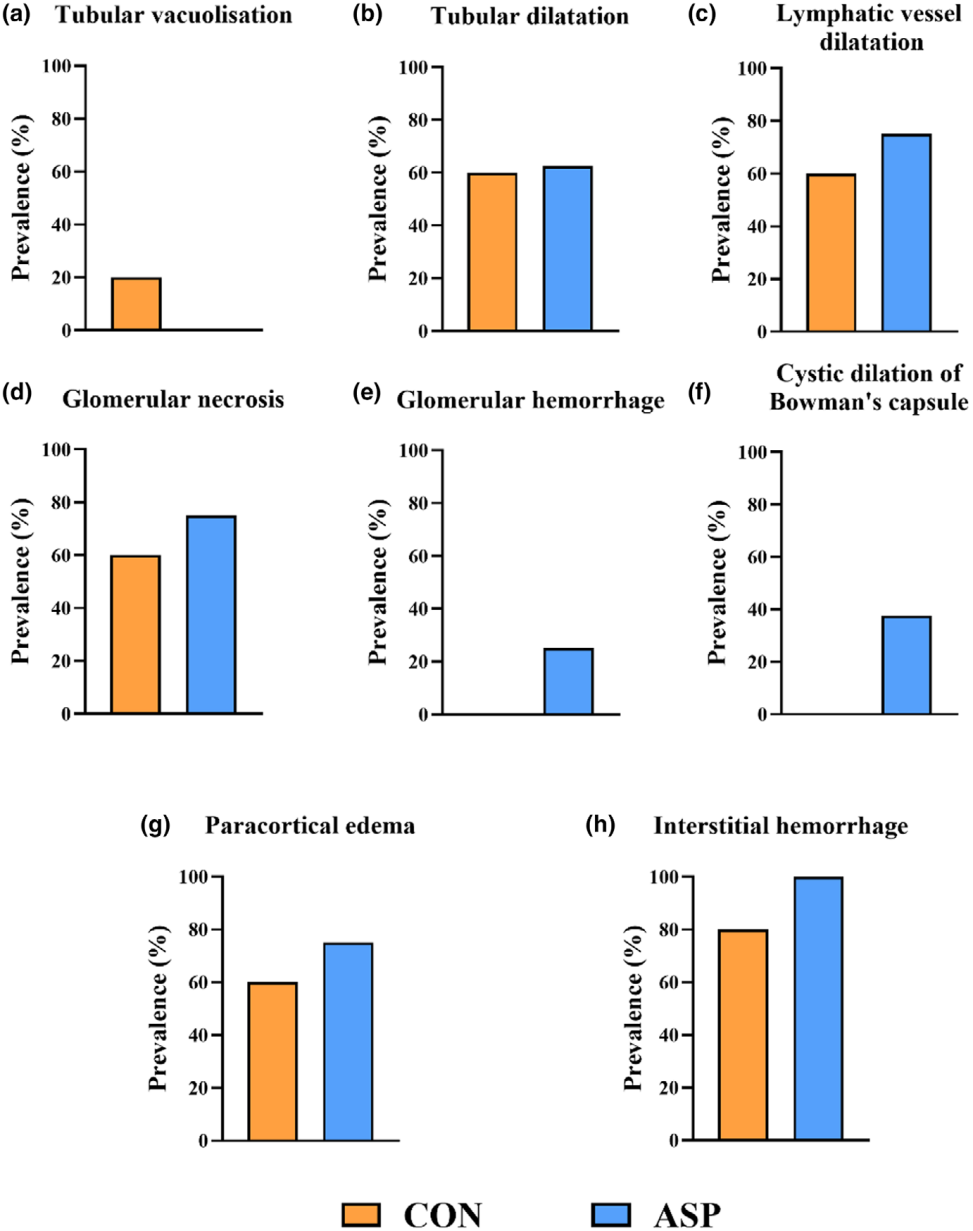
Effects of asphyxia on the prevalence of kidney histological lesions in pigs survived to 24 h. The prevalence of (a and b) tubular vacuolization and dilatation, respectively; (c) Lymphatic vessel dilatation; (d–f) Glomerular necrosis, hemorrhage, and cystic dilation of Bowman's capsule, respectively; (g and h) Paracortical edema and interstitial hemorrhage, respectively. HE kidney sections were used for the pathological evaluation. *n* = 5 for control (CON) and *n* = 8 for asphyxia (ASP).

While of the 47 pigs subjected to asphyxia, we euthanized 13 (27.7%) shortly after birth as we unable to resuscitate them, only two of the 41 pigs (4.9%) in the control group were euthanized within the first 24 h (*p* = 0.005, Figure 2a). Body weight was similar for ASP and CON (Figure 2b). Likewise, kidney weight relative to body weight, and eGFR indexed to kidney weight were similar for ASP and CON, while eGFR indexed to body weight was lower (*p* = 0.035) in ASP versus CON (Figure 2c–e). Plasma creatinine, albumin, total protein, or blood urea nitrogen (BUN) were similar between ASP and CON (Figure 2f–h). Asphyxia increased urine creatinine and potassium (K^+^) levels (both *p* < 0.05), but had no effect on urine total protein, sodium (Na^+^), or chloride (Cl^−^) levels at 24 h (Figure 2j–n). The levels of blood electrolytes during the first 24 h are presented in Figure 3. Briefly, asphyxia led to an immediate decrease in blood Na^+^ and Cl^−^ levels and increase in blood K^+^ and calcium (Ca^2+^) levels (all *p* < 0.01). Electrolyte levels of asphyxiated pigs soon corrected to the levels of control pigs after birth, yet at 24 h, the blood K^+^ and Cl^−^ were lower in ASP versus CON (both *p* < 0.05). Blood gas‐related parameters are shown in Figure S1. Asphyxia caused an immediate increase in blood lactate and pCO_2_, and a decrease in blood pH (all *p* < 0.001). After birth, pH and pCO_2_ quickly normalized in ASP pigs, while blood lactate remained higher for 12 h. Likewise, asphyxia led to an immediate drop in pO_2_ (*p* < 0.001), which however was normalized after birth. Additionally, asphyxia immediately increased blood total hemoglobin (tHb) while decreasing carboxyhemoglobin (COHb) levels (*p* < 0.001). Subsequently, asphyxiated pigs consistently showed lower blood tHb and higher blood COHb levels (*p* < 0.05). As asphyxia associates can cause renal pathological changes, albeit with more pronounced changes in the tubules relative to glomeruli possibly due to the tubules' high metabolic rate (Gerosa et al., 2015; Ikeda et al., 2000), we aimed to comprehensively characterize kidney pathological lesions. We specifically evaluated structural changes in the tubules (including tubular vacuolization and dilatation), glomeruli (including glomerular hemorrhage, necrosis, and cystic dilation of Bowman's capsule), as well as interstitial hemorrhage, paracortical edema, and lymphatic vessel dilatation. Surprisingly, we found no differences in the prevalence of kidney histological lesions in pigs that survived to 24 h (Figure 4). Although this may indicate that short transient asphyxia does not affect histological structures in the kidney, we cannot exclude a possible selection bias due to the large differences in survival between ASP and CON.

### 3.2 The effects of asphyxia and plasma feeding on clinical and paraclinical parameters, blood gas, and renal histological lesions in the 72‐h study

**FIGURE 5:**
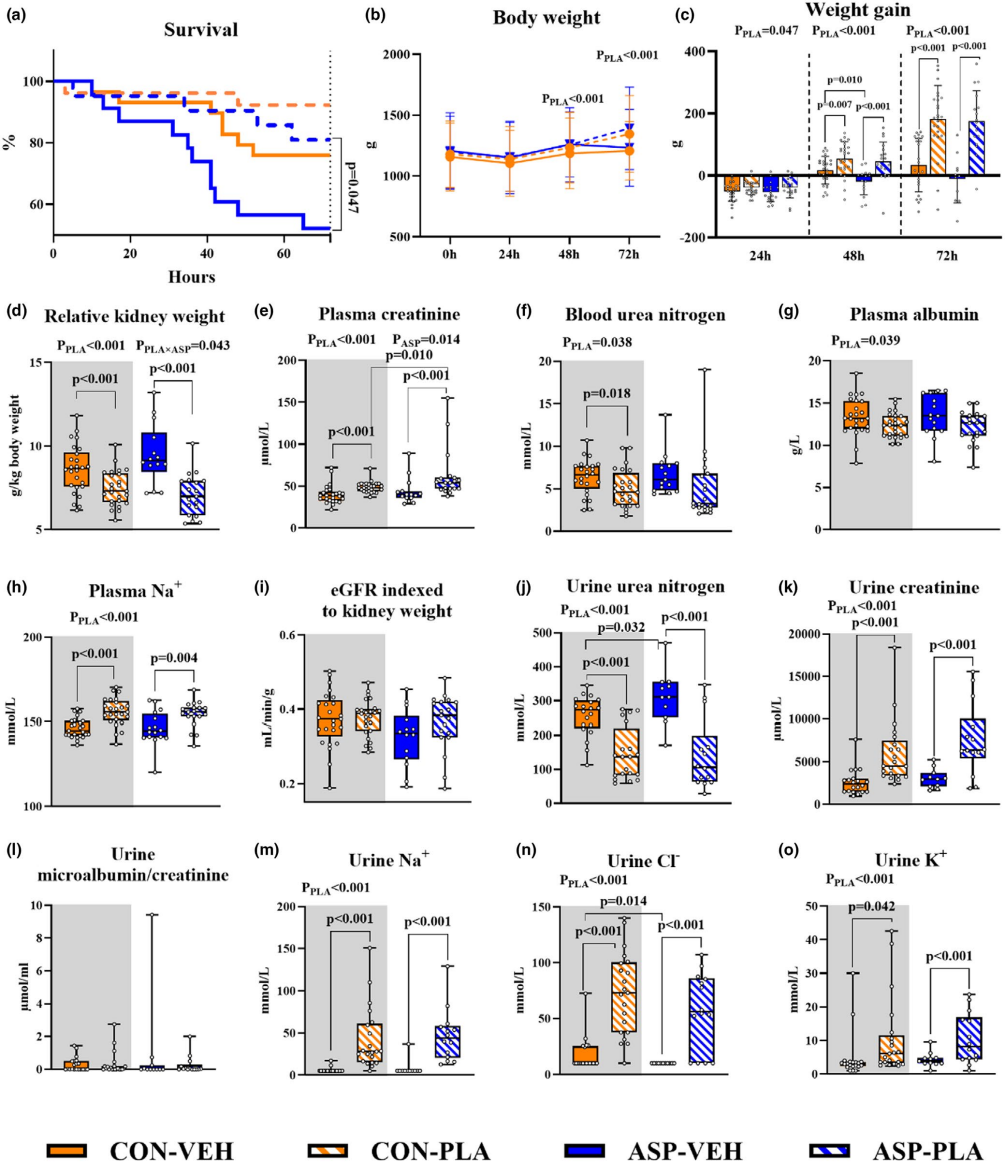
Effects of asphyxia and plasma feeding on clinical outcomes, blood, and urine biochemistry in pigs during the first 72 h. (a–c) The survival rate, body weight, and weight gain. (d) The relative kidney weight. (e–h) Plasma biochemistry. (i) The estimated glomerular filtration rate (eGFR) indexed to kidney weight. (j–o) Urine biochemistry. The sample size (*n*) for each group was as follows: CON‐VEH (*n* = 20–27), CON‐PLA (*n* = 20–25), ASP‐VEH (*n* = 14–20), and ASP‐PLA (*n* = 12–19). *P*_PLA_, *P*_ASP_, and *P*_PLA*ASP_ indicate the significant impacts of plasma feeding, asphyxia and their interaction, respectively, across all animals.

From birth to 72 h, there was a higher survival rate in ASP‐PLA versus ASP‐VEH (89.5% versus 60.0%, *p* = 0.047, Figure 5a). Likewise, in non‐asphyxiated pigs, plasma feeding led to higher survival (96.0% vs. 81.5%). After an initial weight loss during the first 24 h, most pigs, except pigs in ASP‐VEH group, gradually regained and increased their body weight. At 48 h and 72 h, PLA pigs had higher body weight and weight gain than VEH pigs (all *P*_*PLA*_ <0.001, Figure 5b,c). Finally, the ASP‐VEH group showed a tendency towards decreased body weight compared to CON‐VEH at 48 h (*p* = 0.010). As a result of larger body weight, the PLA group showed lower relative kidney weight at 72 h (*P*_PLA_ < 0.001, Figure 5d). Compared to VEH, pigs in the PLA group (Figure 5e–h) showed higher plasma creatinine and Na^+^ levels (both *P*_*PLA*_ < 0.001), but lower BUN and albumin levels (both *P*_*PLA*_ < 0.05), resulting in a lower BUN‐to‐plasma creatinine ratio (*p* < 0.001, Figure S2a). Besides, ASP‐PLA pigs had a tendency towards higher plasma creatinine levels than CON‐PLA (*p* = 0.010). No differences were observed in plasma levels of inorganic phosphate (Pi), K^+^, Mg^2+^, or Ca^2+^ between groups (Figure S2b–e). Iron (Fe^2+^) levels were higher in the ASP‐PLA group compared to the ASP‐VEH group (*p* = 0.029, Figure S2f). No differences were found in eGFR indexed to kidney weight (Figure 5i).

In urine biochemistry (Figure 5j–o), the PLA groups showed higher levels of creatinine, Na^+^, Cl^−^, and K^+^, along with lower levels of urine urea nitrogen relative to the VEH groups (all *P*_*PLA*_ < 0.001). Besides, compared to CON‐VEH, pigs in the ASP‐VEH group had lower urine Cl^−^ and higher urine urea nitrogen (both *p* < 0.05). There was no difference in urine microalbumin‐to‐creatinine ratio between the groups.

The effects of asphyxia and plasma feeding on renal histological changes were independently evaluated by two histopathologists. In the results from evaluator 1 (Figure S3), the ASP‐PLA group had increased prevalence of tubular dilatation than the CON‐PLA group (*p* = 0.011). In the results from evaluator 2 (Figure S4), no evident differences were found.

### 3.3 The effects of asphyxia and plasma feeding on renal gene expression in the 72‐h study

**FIGURE 6:**
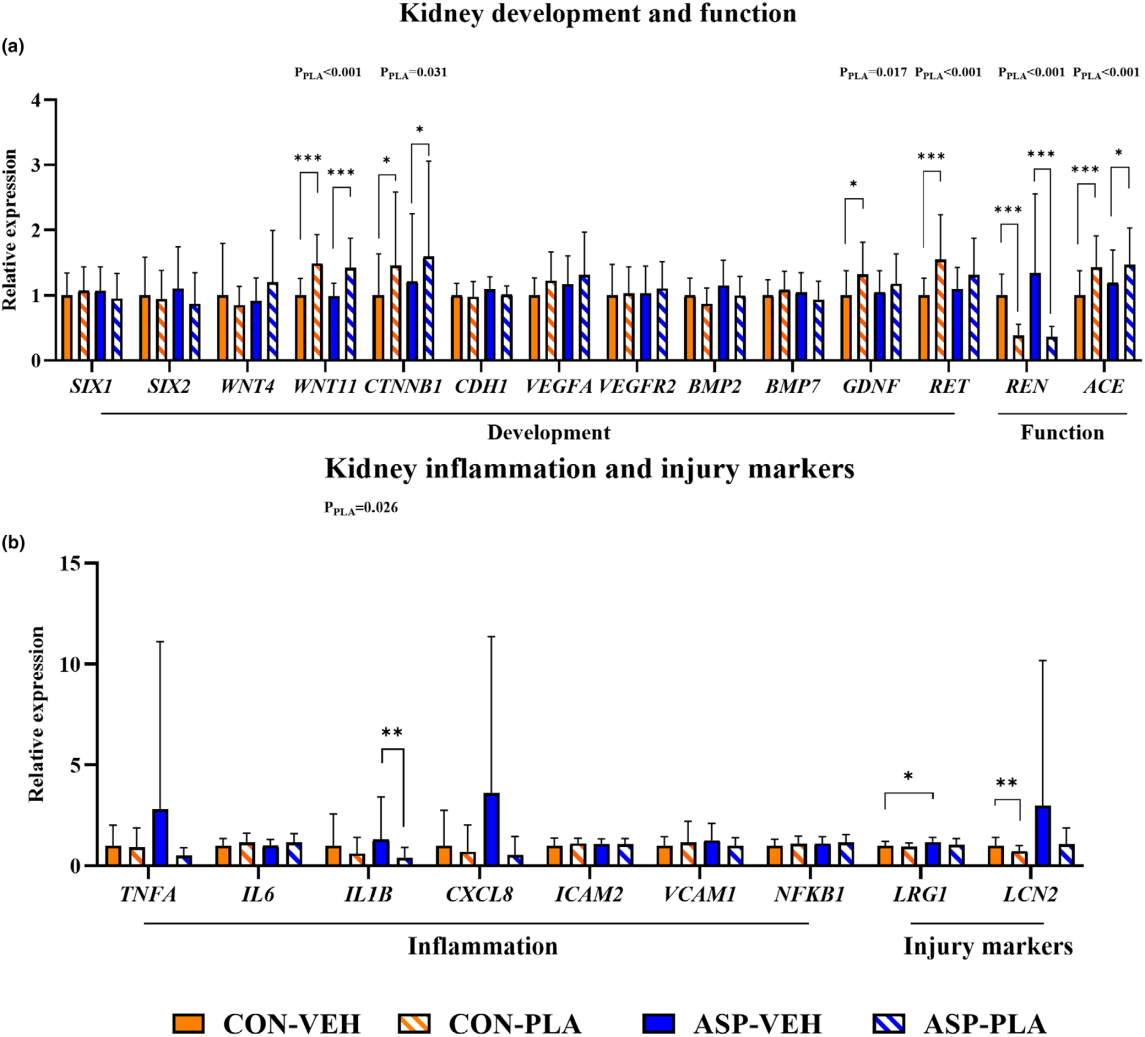
Effects of asphyxia and plasma feeding on renal gene expression related to kidney development, function, inflammation, and injury of pigs in the 72‐h study. (a) The renal gene expression related to kidney development and function. (b) The renal gene expression related to kidney inflammation and injury. All data for CON‐VEH (*n* = 25–27), CON‐PLA (*n* = 25), ASP‐VEH (*n* = 19–20), and ASP‐PLA (*n* = 16–19) are presented as the means ± SD. *p < 0.05, **p < 0.01, ***p < 0.001. *P*_PLA_ indicates the significant impact of plasma feeding across all animals.

**FIGURE 7:**
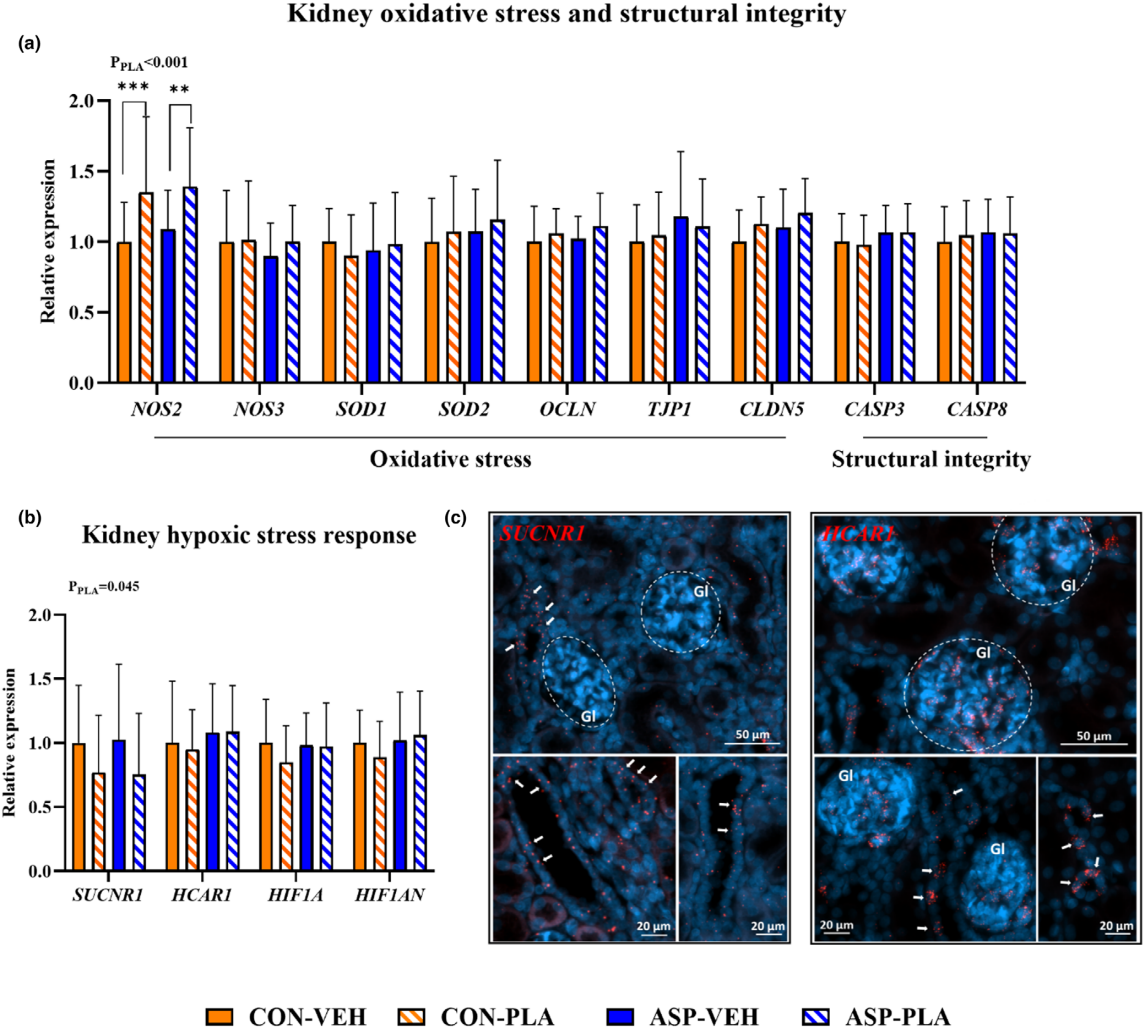
Effects of asphyxia and plasma feeding on pig kidney gene expression related to renal stress responses and structural integrity in the 72‐h study. (a) The kidney gene expression related to oxidative stress and structural integrity. (b) The kidney gene expression related to hypoxic stress response. All data for CON‐VEH (*n* = 25–27), CON‐PLA (*n* = 25), ASP‐VEH (*n* = 19–20), and ASP‐PLA (*n* = 16–19) are presented as the means ± SD. **p < 0.01, and ***p < 0.001. *P*_PLA_ indicates the significant impact of plasma feeding across all animals. (c) Fluorescent RNAscope in situ hybridization illustrates that cells expressing succinate receptor (*SUCNR1*) are localized in the tubular walls but not in the glomerular tuft. The expression of lactate receptor (*HCAR1*) is predominantly localized within glomeruli and in some cells of the proximal tubule.

Relative expression of genes related to kidney development, function, inflammation, and injury are shown in Figure 6a,b. PLA upregulated expression of development‐related genes including wnt family member 11 (*WNT11*), catenin beta 1 (*CTNNB1*), glial cell line‐derived neurotrophic factor (*GDNF*), and Ret proto‐oncogene (*RET*) relative to VEH (all *P*_*PLA*_ < 0.05). Regarding kidney function, PLA led to upregulation of angiotensin I converting enzyme (*ACE*), but downregulation of renin (*REN*) relative to VEH (both *P*_PLA_ <0.001). In the context of asphyxia, PLA led to downregulation of inflammatory genes including interleukin‐1 beta (*IL1B*) (both *p* < 0.01) compared to VEH. ASP‐VEH upregulated leucine rich alpha‐2‐glycoprotein 1 (*LRG1*) compared to CON‐VEH (*p* < 0.05). Furthermore, plasma feeding downregulated lipocalin‐2 (*LCN2*) in the control pigs (*p* < 0.01). For kidney stress response, PLA upregulated nitric oxide synthase 2 (*NOS2*), while downregulating the succinate receptor gene expression (*SUCNR1*) relative to VEH (both *P*_PLA_ < 0.05, Figure 7a,b). Figure 7c illustrates the localization of expressed *SUCNR1* and lactate receptor (*HCAR1*) in the pig kidney using fluorescent RNAscope. Cells expressing *SUCNR1* are detectable in the tubular walls but not in the glomerular tuft. The expression of *HCAR1* is predominantly detected within glomeruli and in some cells of the proximal tubule.

## 4 DISCUSSION

Neonatal asphyxia poses a significant global health challenge for newborns (Lawn et al., 2005). Apart from humans, asphyxia also presents significant challenges in farm animals such as pigs (Alonso‐Spilsbury et al., 2005). The pig and human kidneys are comparable in terms of gross anatomy, physiology, and pathophysiology (Cullen‐McEwen et al., 2016). In this study, we developed a novel neonatal pig asphyxia model by clamping the umbilical cord before delivery, which partly mimics the asphyxia condition encountered in human fetuses during the delivery process. We used this pig model to understand how asphyxia can impact the overall and organ health of newborns, and data on brain and gut effects derived from the same animals as used in the current study are presented elsewhere (Nordsten et al., 2024; Ventura et al., 2024). For asphyxiated full‐term infants, therapeutic hypothermia (TH) after birth is currently used as a standard treatment with the main purpose to mitigate the progression of hypoxic–ischemic encephalopathy (HIE). Although TH substantially lowers the risk of adverse outcomes in cases of moderate‐to‐severe HIE, neurological impairments persist in up to 40% of survivors (Ventura et al., 2024). Moreover, TH was found to potentially reduce the incidence of AKI among term neonates with perinatal asphyxia (Tanigasalam et al., 2016). Nevertheless, AKI remains a common complication of perinatal asphyxia despite treatment with TH (Bozkurt & Yucesoy, 2021; Selewski et al., 2013). Hence, exploring alternative treatment modalities may be relevant. In this study, we specifically examined plasma feeding as a potential intervention for asphyxiated newborns, focusing on its role as a nutritional strategy—a relatively underexplored area.

Using this innovative asphyxia pig model, we first demonstrated how asphyxia caused by umbilical cord compression can impact newborn kidney function and overall health. At 24 h after asphyxia, only minor eGFR changes were found in surviving pigs. In pigs sampled between 24 and 72 h, no changes in eGFR were observed. Whereas this may reflect a selection bias, as many unsuccessfully resuscitated pigs could not be sampled, other studies have demonstrated that there were only minor or no changes in GFR in newborn piglets (6–96 h of age) and infants (within the first 24 h) shortly after asphyxia (Alward et al., 1978; Lubis et al., 2001). This evidence suggests that renal dysfunction and impairments may be difficult to detect in the early life of asphyxiated newborns. However, our gene expression data from the 72‐h study indicated renal injury and inflammation may potentially manifest in asphyxiated pigs.

Asphyxia is known to disrupt electrolyte and fluid balance, leading to reduced blood Na^+^ and increased blood K^+^ in infants (Basu et al., 2010; Gupta & Bora, 2021; Thakur et al., 2018), and we found similar electrolyte changes in the asphyxiated pigs. Na^+^ and K^+^ are the major cations of extracellular and intracellular fluid, respectively (Pohl et al., 2013). The normal membrane potential is maintained through the activity of Na^+^/K^+^‐ATPase, which transports Na^+^ extracellularly and K^+^ intracellularly. During episodes of asphyxia, mitochondrial oxidative phosphorylation, which is essential for ATP production to maintain membrane potential, becomes compromised. This impairment leads to dysfunctional Na^+^/K^+^‐ATPase activity, potentially resulting in an imbalance of Na^+^ and K^+^. Interestingly, we also observed higher blood Ca^2+^ levels immediately after asphyxia, contrary to clinical observations (Bahatkar & Aundhakar, 2021; Basu et al., 2010; Injeti et al., 2021), which may indicate differences in Ca^2+^ regulatory mechanisms between pigs and humans in a post‐asphyxia state. Furthermore, we showed that the electrolyte changes observed immediately after birth in asphyxiated pigs were rapidly corrected after 4 h, indicating that the electrolyte imbalance after this type of asphyxia is transient. Moreover, a lower blood K^+^ from 9 to 24 h, and a higher urine K^+^ at 24 h in asphyxiated pigs might indicate decreased reabsorption of K^+^ in the kidney tubules.

Body fluid homeostasis is particularly important in the immediate postnatal phase where large and acute circulatory changes take place. Immediately after birth, there is a normal and necessary process of water loss in newborns (Mulder & Gardner, 2015), and we consistently observe this loss in newborn pigs within the first 24 h. From 24 to 72 h, there was a gradual increase in body weight in most pigs, which represents both lean body mass accretion and body hydration. The increase in body weight was not observed in the ASP‐VEH group, suggesting that asphyxia may impair body hydration, potentially due to impaired renal tubular reabsorption of water following asphyxia (Rai & Singh, 2016; Stonestreet et al., 1984). Breastfeeding plays an important role in postnatal body hydration in both humans and pigs (McCance & Widdowson, 1959; Mulder & Gardner, 2015). Interestingly, PLA stimulated higher body water retention compared with VEH as indicated by higher body weight, lower plasma albumin and BUN levels, decreased BUN‐to‐creatinine ratio (Baum et al., 1975), down‐regulated renal expression of *REN* (responsible for restoration of blood pressure during vascular volume depletion (MacGregor et al., 1981)) and more condensed urine in PLA relative to VEH. As Na^+^ levels in the PLA diet were substantially higher than the VEH diet, we speculate that the increased water retention in plasma‐fed pigs resulted partly from higher intake of Na^+^, leading to higher blood osmolarity after feeding. Plasma feeding potentially promoted renal health compared to vehicle feeding. This is mainly indicated by the upregulation of several key genes related to kidney development (Costantini & Shakya, 2006; Faa et al., 2012; Wang et al., 2018), and downregulation of certain genes related to renal inflammation after asphyxia. Importantly, a study showed that restricting fluid and Na^+^ intake in term asphyxiated newborns treated with hypothermia, led to a higher incidence of kidney injury (La Haye‐Caty et al., 2020), indicating that maintaining Na^+^ intake and water retention might be important for kidney health in asphyxiated newborns. However, a potential risk of plasma feeding in newborns is the intake of excessive nutrients and other substances. For instance, we observed higher plasma creatinine levels in piglets fed plasma‐based diets, which might result from their intake of maternal plasma creatinine. The creatinine level in the sow plasma used in this study could have been elevated beyond physiological levels, as zolazepam is known to cause a post‐anesthesia increase in serum creatinine levels (Karasu et al., 2018).

In conclusion, using a novel neonatal pig asphyxia model, this study demonstrates that birth asphyxia may lead to minor renal dysfunction, and consequently electrolyte imbalance and compromised postnatal fluid retention during the first 72 h. Plasma feeding may improve fluid retention and kidney health in piglets partly via providing substantial Na^+^. This study has certain limitations and perspectives: Future studies could be designed with more direct monitoring of in vivo GFR and urine output. Further, plasma feeding intervention could be combined with other standard treatments such as TH in the pig model for improved clinical relevance. Despite these limitations, our study has provided novel information regarding the effects of plasma feeding on postnatal hydration and kidney protection in neonates following asphyxia. This can serve as a good reference for further refining and applying plasma feeding in both asphyxiated human newborns and neonatal farm production animals.

## AUTHOR CONTRIBUTIONS

Conception and design: PTS, SP, DNN, TT, and JZ; data acquisition: JZ, SP, RD, CKF, KS, HEJ, and TT; data analysis: JZ; data interpretation: all co‐authors; writing original draft: JZ; critical review and editing: JZ and TT; approval of the final manuscript: all co‐authors.

## FUNDING INFORMATION

The study was partly supported by funding from the Carlsberg Foundation (CF21‐0536; grant recipient: Stanislava Pankratova). Author JZ's salary was financed by China Scholarship Council (202006910004). No other financial assistance was received in support of this study.

## CONFLICT OF INTEREST STATEMENT

None declared.

## CONSENT

Patient consent was not required.

## ETHICS STATEMENT

The animal experimental protocol was approved by the Danish Animal Experiments Inspectorate (licence no.: 2020–15‐0201–00520), which is in accordance with the guidelines from Directive 2010/63/EU of the European Parliament.

## Supporting information


Appendix S1.

## Data Availability

All datasets generated during and analyzed during the current study, including raw data used for all figures and analysis, are available from the corresponding author on reasonable request.

## References

[phy270238-bib-0001] Agras, P. I. , Tarcan, A. , Baskin, E. , Cengiz, N. , Gürakan, B. , & Saatci, U. (2004). Acute renal failure in the neonatal period. Renal Failure, 26, 305–309. 15354981 10.1081/jdi-200026749

[phy270238-bib-0002] Alonso‐Spilsbury, M. , Mota‐Rojas, D. , Villanueva‐García, D. , Martínez‐Burnes, J. , Orozco, H. , Ramírez‐Necoechea, R. , Mayagoitia, A. L. , & Trujillo, M. E. (2005). Perinatal asphyxia pathophysiology in pig and human: A review. Animal Reproduction Science, 90, 1–30. 16257594 10.1016/j.anireprosci.2005.01.007

[phy270238-bib-0003] Alward, C. T. , Hook, J. B. , Helmrath, T. A. , & Bailie, M. D. (1978). Effects of asphyxia on renal function in the newborn piglet. Pediatric Research, 12, 225–228. 580450 10.1203/00006450-197803000-00013

[phy270238-bib-0004] Antonucci, R. , Porcella, A. , & Pilloni, M. D. (2014). Perinatal asphyxia in the term newborn. Journal of Pediatric and Neonatal Individualized Medicine, 3, e030269.

[phy270238-bib-0005] Bahatkar, K. , & Aundhakar, C. (2021). Electrolyte status and plasma glucose levels in birth asphyxia: A case–control study. Journal of Medical Sciences, 41, 17–21.

[phy270238-bib-0006] Balan, P. , Staincliffe, M. , & Moughan, P. J. (2021). Effects of spray‐dried animal plasma on the growth performance of weaned piglets—A review. Journal of Animal Physiology and Animal Nutrition, 105, 699–714. 32860645 10.1111/jpn.13435

[phy270238-bib-0007] Basu, P. , Som, S. , Das, H. , & Choudhuri, N. (2010). Electrolyte status in birth asphyxia. The Indian Journal of Pediatrics, 77, 259–262. 20177828 10.1007/s12098-010-0034-0

[phy270238-bib-0008] Baum, N. , Dichoso, C. C. , & Carlton, C. E. (1975). Blood urea nitrogen and serum creatinine: Physiology and interpretations. Urology, 5, 583–588. 1093306 10.1016/0090-4295(75)90105-3

[phy270238-bib-0009] Behrman, R. , Lees, M. , Peterson, E. , de Lannoy, C. W. , & Seeds, A. (1970). Distribution of the circulation in the Normal and asphyxiated fetal primate. American Journal of Obstetrics and Gynecology, 108, 956–969. 4992043 10.1016/0002-9378(70)90341-8

[phy270238-bib-0010] Bonnitcha, P. , Grieve, S. , & Figtree, G. (2018). Clinical imaging of hypoxia: Current status and future directions. Free Radical Biology & Medicine, 126, 296–312. 30130569 10.1016/j.freeradbiomed.2018.08.019

[phy270238-bib-0011] Bozkurt, O. , & Yucesoy, E. (2021). Acute kidney injury in neonates with perinatal asphyxia receiving therapeutic hypothermia. American Journal of Perinatology, 38, 922–929. 31986537 10.1055/s-0039-1701024

[phy270238-bib-0012] Cargill, K. R. , Chiba, T. , Murali, A. , Mukherjee, E. , Crinzi, E. , & Sims‐Lucas, S. (2020). Prenatal hypoxia increases susceptibility to kidney injury. PLoS One, 15, e0229618. 32084244 10.1371/journal.pone.0229618 PMC7034911

[phy270238-bib-0013] Cohn, H. E. , Sacks, E. J. , Heymann, M. A. , & Rudolph, A. M. (1974). Cardiovascular Responses to Hypoxemia and Acidemia in Fetal Lambs. American Journal of Obstetrics and Gynecology, 120, 817–824. 4429091 10.1016/0002-9378(74)90587-0

[phy270238-bib-0014] Costantini, F. , & Shakya, R. (2006). Gdnf/ret signaling and the development of the kidney. BioEssays, 28, 117–127. 16435290 10.1002/bies.20357

[phy270238-bib-0015] Cullen‐McEwen, L. , Sutherland, M. R. , & Black, M. J. (2016). Kidney development, disease, repair and regeneration (pp. 27–40). Elsevier.

[phy270238-bib-0016] Durkan, A. M. , & Alexander, R. T. (2011). Acute kidney injury post neonatal asphyxia. The Journal of Pediatrics, 158, e29–e33. 21238703 10.1016/j.jpeds.2010.11.010

[phy270238-bib-0017] Faa, G. , Gerosa, C. , Fanni, D. , Monga, G. , Zaffanello, M. , van Eyken, P. , & Fanos, V. (2012). Morphogenesis and molecular mechanisms involved in human kidney development. Journal of Cellular Physiology, 227, 1257–1268. 21830217 10.1002/jcp.22985

[phy270238-bib-0018] Gallo, D. , de Bijl‐Marcus, K. A. , Alderliesten, T. , Lilien, M. , & Groenendaal, F. (2021). Early acute kidney injury in preterm and term neonates: Incidence, outcome, and associated clinical features. Neonatology, 118, 174–179. 33780939 10.1159/000513666

[phy270238-bib-0019] Gasthuys, E. , Devreese, M. , Millecam, J. , Sys, S. , Vanderperren, K. , Delanghe, J. , Vande Walle, J. , Heyndrickx, M. , & Croubels, S. (2017). Postnatal maturation of the glomerular filtration rate in conventional growing piglets as potential juvenile animal model for preclinical pharmaceutical research. Frontiers in Pharmacology, 8, 431. 28706488 10.3389/fphar.2017.00431 PMC5489626

[phy270238-bib-0020] Gerosa, C. , Iacovidou, N. , Argyri, I. , Fanni, D. , Papalois, A. , Aroni, F. , Faa, G. , Xanthos, T. , & Fanos, V. (2015). Histopathology of renal asphyxia in newborn piglets: Individual susceptibility to tubular changes. World Journal of Nephrology, 4, 313–318. 25949946 10.5527/wjn.v4.i2.313 PMC4419142

[phy270238-bib-0021] Gupta, J. , & Bora, B. (2021). A study of serum electrolytes level in newborns with birth asphyxia. International Journal Dental and Medical Sciences Research, 3, 265–268.

[phy270238-bib-0022] Hassiotou, F. , & Geddes, D. (2013). Anatomy of the human mammary gland: Current status of knowledge. Clinical Anatomy, 26, 29–48. 22997014 10.1002/ca.22165

[phy270238-bib-0023] Ikeda, T. , Murata, Y. , Quilligan, E. J. , Parer, J. T. , Murayama, T. , & Koono, M. (2000). Histologic and biochemical study of the brain, heart, kidney, and liver in asphyxia caused by occlusion of the umbilical cord in near‐term fetal lambs. American Journal of Obstetrics and Gynecology, 182, 449–457. 10694351 10.1016/s0002-9378(00)70238-9

[phy270238-bib-0024] Injeti, G. , Kher, A. , Taksande, A. , & Panwar, A. S. (2021). Determination of serum electrolyte and calcium abnormalities in neonates with birth asphyxia. Journal of Pharmaceutical Research International, 33, 807–815.

[phy270238-bib-0025] Jhajra, S. , Nanda, D. , & Dalal, J. S. (2024). Enteral feeding in neonates≥ 34 weeks of gestation with moderate to severe birth asphyxia: A retrospective observational study. Tropical Doctor, 54(4), 312–316. 10.1177/00494755241255162 38767174

[phy270238-bib-0026] Karasu, A. , Altug, N. , Aslan, L. , Bakir, B. , & Yüksek, N. (2018). Evaluation of the anesthetic effects of xylazine‐ketamine, xylazine‐tiletamine‐zolazepam and tiletamine‐zolazepam using clinical and laboratory parameters in rabbits. Medycyna Weterynaryjna‐Veterinary Medicine‐Science and Practice, 74, 646–652.

[phy270238-bib-0027] Kritzinger, A. , Krüger, E. , & Pottas, L. (2017). Breastfeeding and swallowing in a neonate with mild hypoxic‐ischaemic encephalopathy. The South African Journal of Communication Disorders, 64, 1–7. 10.4102/sajcd.v64i1.209 PMC5843037 28582997

[phy270238-bib-0028] Krüger, E. , Kritzinger, A. , & Pottas, L. (2019). Oropharyngeal dysphagia in breastfeeding neonates with hypoxic‐ischemic encephalopathy on therapeutic hypothermia. Breastfeeding Medicine, 14, 718–723. 31532260 10.1089/bfm.2019.0048

[phy270238-bib-0029] La Haye‐Caty, N. , Barbosa Vargas, S. , Maluorni, J. , Rampakakis, E. , Zappitelli, M. , & Wintermark, P. (2020). Impact of restricting fluid and sodium intake in term asphyxiated newborns treated with hypothermia. The Journal of Maternal‐Fetal & Neonatal Medicine, 33, 3521–3528. 30714445 10.1080/14767058.2019.1578747

[phy270238-bib-0030] Lacombe, C. , da Silva, J. L. , Bruneval, P. , Fournier, J. G. , Wendling, F. , Casadevall, N. , Camilleri, J. P. , Bariety, J. , Varet, B. , & Tambourin, P. (1988). Peritubular cells are the site of erythropoietin synthesis in the murine hypoxic kidney. The Journal of Clinical Investigation, 81, 620–623. 3339134 10.1172/JCI113363 PMC329613

[phy270238-bib-0031] Lawn, J. E. , Cousens, S. , Zupan, J. , & Lancet Neonatal Survival Steering Team . (2005). 4 million neonatal deaths: When? Where? Why? Lancet, 365, 891–900. 15752534 10.1016/S0140-6736(05)71048-5

[phy270238-bib-0032] Lubis, E. N. , Yanda, S. , Akbar, K. , Tjipta, G. D. , & Aldy, D. (2001). The effect of neonatal asphyxia on renal function. Paediatrica Indonesiana, 41, 175–179.

[phy270238-bib-0033] MacGregor, G. , Markandu, N. , Roulston, J. , Jones, J. , & Morton, J. (1981). Maintenance of blood pressure by the renin–angiotensin system in normal man. Nature, 291, 329–331. 7015149 10.1038/291329a0

[phy270238-bib-0034] McCance, R. , & Widdowson, E. M. (1959). The effect of colostrum on the composition and volume of the plasma of new‐born piglets. The Journal of Physiology, 145, 547. 13642319 10.1113/jphysiol.1959.sp006160 PMC1356960

[phy270238-bib-0035] Moshiro, R. , Mdoe, P. , & Perlman, J. M. (2019). A global view of neonatal asphyxia and resuscitation. Frontiers in Pediatrics, 7, 489. 31850287 10.3389/fped.2019.00489 PMC6902004

[phy270238-bib-0036] M'Rabet, L. , Vos, A. P. , Boehm, G. , & Garssen, J. (2008). Breast‐feeding and its role in early development of the immune system in infants: Consequences for health later in life. Journal of Nutrition, 138, 1782S–1790S. 18716187 10.1093/jn/138.9.1782S

[phy270238-bib-0037] Mulder, P. J. , & Gardner, S. E. (2015). The healthy newborn hydration model: A new model for understanding newborn hydration immediately after birth. Biological Research for Nursing, 17, 94–99. 25504955 10.1177/1099800414529362

[phy270238-bib-0038] Nordsten, M. J. B. , Winther, C. L. , Haugaard, M. M. , Skovgaard, K. , Thymann, T. , & Sangild, P. T. (2024). Enteral plasma feeding improves gut function and immunity in piglets after birth asphyxia. Pediatric Research. 10.1038/s41390-024-03376-0. Online ahead of print. PMC12014487 39034356

[phy270238-bib-0039] Okazaki, K. , Nakamura, S. , Koyano, K. , Konishi, Y. , Kondo, M. , & Kusaka, T. (2023). Neonatal asphyxia as an inflammatory disease: Reactive oxygen species and cytokines. Frontiers in Pediatrics, 11, 1070743. 36776908 10.3389/fped.2023.1070743 PMC9911547

[phy270238-bib-0040] Perrone, S. , Mussap, M. , Longini, M. , Fanos, V. , Bellieni, C. V. , Proietti, F. , Cataldi, L. , & Buonocore, G. (2007). Oxidative kidney damage in preterm newborns during perinatal period. Clinical Biochemistry, 40, 656–660. 17320066 10.1016/j.clinbiochem.2007.01.012

[phy270238-bib-0041] Pohl, H. R. , Wheeler, J. S. , & Murray, H. E. (2013). Sodium and potassium in health and disease. Interrelations Between Essential Metal Ions and Human Diseases, 13, 29–47. 10.1007/978-94-007-7500-8_2 24470088

[phy270238-bib-0042] Rai, S. , & Singh, N. (2016). Renal status (Bu, Uo) in birth asphyxia. International Journal of Contemporary Medical Research, 3, 1428–1430.

[phy270238-bib-0043] Sanchez‐Salcedo, J. , Bonilla‐Jaime, H. , Lozano, M. G. , Hernandez‐Arteaga, S. , Greenwell‐Beare, V. , Vega‐Manriquez, X. , Gonzalez‐Hernandez, M. , & Orozco‐Gregorio, H. (2019). Therapeutics of neonatal asphyxia in production animals: A review. Veterinární Medicína, 64, 191–203.

[phy270238-bib-0044] Sangild, P. T. , Thymann, T. , Schmidt, M. , Stoll, B. , Burrin, D. G. , & Buddington, R. K. (2013). Invited review: The preterm pig as a model in pediatric gastroenterology. Journal of Animal Science, 91, 4713–4729. 23942716 10.2527/jas.2013-6359 PMC3984402

[phy270238-bib-0045] Selewski, D. T. , Jordan, B. K. , Askenazi, D. J. , Dechert, R. E. , & Sarkar, S. (2013). Acute kidney injury in asphyxiated newborns treated with therapeutic hypothermia. The Journal of Pediatrics, 162, 725–729. 23149172 10.1016/j.jpeds.2012.10.002

[phy270238-bib-0046] Shu, S. , Wang, Y. , Zheng, M. , Liu, Z. , Cai, J. , Tang, C. , & Dong, Z. (2019). Hypoxia and hypoxia‐inducible factors in kidney injury and repair. Cells, 8, 207. 30823476 10.3390/cells8030207 PMC6468851

[phy270238-bib-0047] Siegel, S. R. (1982). Hormonal and renal interaction in body fluid regulation in the newborn infant. Clinics in Perinatology, 9, 535–557. 6761037

[phy270238-bib-0048] Skovgaard, K. , Cirera, S. , Vasby, D. , Podolska, A. , Breum, S. Ø. , Dürrwald, R. , Schlegel, M. , & Heegaard, P. M. H. (2013). Expression of innate immune genes, proteins and Micrornas in lung tissue of pigs infected experimentally with influenza virus (H1n2). Innate Immunity, 19, 531–544. 23405029 10.1177/1753425912473668

[phy270238-bib-0049] Stonestreet, B. S. , Laptook, A. R. , Siegel, S. R. , & Oh, W. (1984). The renal response to acute asphyxia in spontaneously breathing newborn lambs. Early Human Development, 9, 347–361. 6378587 10.1016/0378-3782(84)90079-3

[phy270238-bib-0050] Stuebe, A. (2009). The risks of not breastfeeding for mothers and infants. Reviews in Obstetrics and Gynecology, 2, 222–231. 20111658 PMC2812877

[phy270238-bib-0051] Tanigasalam, V. , Bhat, V. , Adhisivam, B. , & Sridhar, M. (2016). Does therapeutic hypothermia reduce acute kidney injury among term neonates with perinatal asphyxia?–a randomized controlled trial. The Journal of Maternal‐Fetal & Neonatal Medicine, 29, 2545–2548. 26456813 10.3109/14767058.2015.1094785

[phy270238-bib-0052] Thakur, J. , Bhatta, N. K. , Singh, R. R. , Poudel, P. , Lamsal, M. , & Shakya, A. (2018). Prevalence of electrolyte disturbances in perinatal asphyxia: A prospective study. Italian Journal of Pediatrics, 44, 1–6. 29784025 10.1186/s13052-018-0496-7 PMC5963047

[phy270238-bib-0053] Truchet, S. , & Ollivier‐Bousquet, M. (2009). Mammary gland secretion: Hormonal coordination of endocytosis and exocytosis. Animal, 3, 1733–1742. 22443558 10.1017/S1751731109990589

[phy270238-bib-0054] Ventura, G. C. , Dyshliuk, N. , Dmytriyeva, O. , Nordsten, M. J. B. , Haugaard, M. M. , Christiansen, L. I. , Thymann, T. , Sangild, P. T. , & Pankratova, S. (2024). Enteral plasma supports brain repair in newborn pigs after birth asphyxia. Brain, Behavior, and Immunity, 119, 693–708. 38677626 10.1016/j.bbi.2024.04.032

[phy270238-bib-0055] Victora, C. G. , Bahl, R. , Barros, A. J. , França, G. V. , Horton, S. , Krasevec, J. , Murch, S. , Sankar, M. J. , Walker, N. , Rollins, N. C. , & Lancet Breastfeeding Series Group . (2016). Breastfeeding in the 21st century: Epidemiology, mechanisms, and lifelong effect. Lancet, 387, 475–490. 26869575 10.1016/S0140-6736(15)01024-7

[phy270238-bib-0056] Wagner, C. L. , Eicher, D. J. , Katikaneni, L. D. , Barbosa, E. , & Holden, K. R. (1999). The use of hypothermia: A role in the treatment of neonatal asphyxia? Pediatric Neurology, 21, 429–443. 10428427 10.1016/s0887-8994(99)00020-x

[phy270238-bib-0057] Walker, W. A. (2004). The dynamic effects of breastfeeding on intestinal development and host defense. Protecting Infants Through Human Milk: Advancing The Scientific Evidence, 554, 155–170. 10.1007/978-1-4757-4242-8_15 15384575

[phy270238-bib-0058] Wang, Y. , Zhou, C. J. , & Liu, Y. (2018). Wnt signaling in kidney development and disease. Progress in Molecular Biology and Translational Science, 153, 181–207. 29389516 10.1016/bs.pmbts.2017.11.019 PMC6008255

[phy270238-bib-0059] Weström, B. , Arévalo Sureda, E. , Pierzynowska, K. , Pierzynowski, S. G. , & Pérez‐Cano, F.‐J. (2020). The immature gut barrier and its importance in establishing immunity in newborn mammals. Frontiers in Immunology, 11, 1153. 32582216 10.3389/fimmu.2020.01153 PMC7296122

