# Supplementary material for: Postnatal enteral plasma supplementation following birth asphyxia increases fluid retention and kidney health in newborn pigs
Source: Physiol Rep. 2025 Feb 5;13(3):e70238. doi: 10.14814/phy2.70238 (PMC11798866; doi:10.14814/phy2.70238)

**Supplemental Figures**

**Postnatal enteral plasma supplementation following birth asphyxia increases fluid retention and kidney health in newborn pigs**

Jingren Zhong^1^, Stanislava Pankratova^1^, Richard Doughty^2^, Christoffer Kirkelund Flyger^3^, Per Torp Sangild^1,4,5^, Kerstin Skovgaard^6^, Henrik Elvang Jensen^3^, Duc Ninh Nguyen^1^, and Thomas Thymann^1 *^

**Affiliations:**

1. Section for Comparative Pediatrics and Nutrition, Department of Veterinary and Animal Sciences, University of Copenhagen, Frederiksberg, Denmark
2. Department of Pathology, Akershus University Hospital, Lørenskog, Norway
3. Section for Pathobiological Sciences, Department of Veterinary and Animal Sciences, University of Copenhagen, Frederiksberg, Denmark
4. Department of Pediatrics, Odense University Hospital, Odense, Denmark
5. Department of Neonatology, Rigshospitalet, Copenhagen, Denmark
6. Department of Biotechnology and Biomedicine, Technical University of Denmark, Lyngby, Denmark

***Correspondence:** Thomas Thymann, Section for Comparative Pediatrics and Nutrition, Department of Veterinary and Animal Sciences, Faculty of Health and Medical Sciences, University of Copenhagen, Dyrlægevej 68, DK-1870 Frederiksberg C, Denmark, Tel: + 45 35 33 26 22, email: [thomas.thymann@sund.ku.dk](mailto:thomas.thymann@sund.ku.dk), ORCID: <https://orcid.org/0000-0001-7480-6064>.

Email address:

Jingren Zhong: [hxn665@sund.ku.dk](mailto:hxn665@sund.ku.dk); Stanislava Pankratova: [stasya@sund.ku.dk](mailto:stasya@sund.ku.dk); Richard Doughty: [rdou@ous-hf.no](mailto:rdou@ous-hf.no); Christoffer Kirkelund Flyger: chkf@sund.ku.dk; Per Torp Sangild: [pts@sund.ku.dk](mailto:pts@sund.ku.dk); Kerstin Skovgaard: [kesk@dtu.dk](mailto:kesk@dtu.dk); Henrik Elvang Jensen: [elvang@sund.ku.dk](mailto:elvang@sund.ku.dk); Duc Ninh Nguyen: [dnn@sund.ku.dk](mailto:dnn@sund.ku.dk).

**Key words:** neonates, asphyxia, plasma, kidneys, electrolytes, fluid

**Supplemental Table S1. Enteral diets information.**

| Product (per L) | Diet A1: | Diet B1: | Diet A2: | Diet B2: |
| --- | --- | --- | --- | --- |
|  | Diets in the first 24h | | Diets from 24h to 72h | |
| Content | Colostrum  + plasma | Colostrum  + WPC/WOI  + water | Formula  + plasma | Formula  + water |
| SHS Liquigen MCT, g |  |  | 70 | 70 |
| Nutricia Calogen LCT, g |  |  | 20 | 20 |
| Fantomalt, g |  |  | 30 | 30 |
| DI-9224/WPC/WOI 90, g |  | 70 | 30 | 105 |
| Phlexy Vits, g | 2 | 2 | 2 | 2 |
| Bovine colostrum powder, g | 100 | 100 |  |  |
| Variolac 855, g |  |  | 10 | 10 |
| Sow plasma, ml | 1000 |  | 955 |  |
| Water, ml |  | 1000 |  | 955 |
| Nutrients composition (per L) | |  |  |  |
| Energy, kJ | 3365 | 3343 | 3866 | 3824 |
| Protein, g | 130 | 130 | 94 | 93 |
| Whey protein, g | 43 | 113 | 27 | 93 |
| Carbohydrate, g | 10 | 10 | 37 | 37 |
| Sugars, g | 10 | 10 | 11 | 11 |
| Fat, g | 27 | 27 | 45 | 45 |
| Sat. fat, g |  |  | 34 | 34 |
| Mono-unsat. Fat |  |  | 6 | 6 |
| Poly-unsat. fat |  |  | 3 | 3 |
| g MCT (rest is LCT) |  |  | 35 | 35 |
| Na, mg | 3320 | 522 | 3375 | 696 |
| K, mg | 172 | 1044 | 557 | 1372 |
| Ca, mg | 91 | 132 | 126 | 164 |
| P, mg | 86 | 160 | 144 | 212 |
| Mg, mg | 19 | 25 | 18 | 24 |
| Fe, mg | 921 | 0 | 880 | 0 |

**Supplemental Table S2. Primer list for Fluidigm.**

| Gene name | Gene symbol | Sequence (5' to 3') | Sequence (3' to 5') |
| --- | --- | --- | --- |
| GAPDH | Glyceraldehyde-3-phosphate dehydrogenase | ACCCAGAAGACTGTGGATGG | AAGCAGGGATGATGTTCTGG |
| HPRT1 | Hypoxanthine phosphoribosyl-transferase I | ACACTGGCAAAACAATGCAA | TGCAACCTTGACCATCTTTG |
| RPL13A | Ribosomal protein L13a | ATTGTGGCCAAGCAGGTACT | AATTGCCAGAAATGTTGATGC |
| PPIA | peptidylprolyl isomerase A (cyclophilin A) | CAAGACTGAGTGGTTGGATGG | TGTCCACAGTCAGCAATGGT |
| YWHAZ | Tyrosine 3-monooxygenase/tryptophan 5-monooxygenase | GCTGCTGGTGATGATAAGAAGG | AGTTAAGGGCCAGACCCAAT |
| SAA | Serum Amyloid A | TGGAGAGCCTACTCGGACAT | CCTTTGGGCAGCATCATAGT |
| TNFA | Tumor Necrosis Factor alpha | CACGTTGTAGCCAATGTCAAAG | GAGGTACAGCCCATCTGTCG |
| CTNNB1 | Catenin Beta 1 | CCAGGATGATCCCAGCTATC | CCCATCAACTGGATAGTCAGC |
| NOS3 | Nitric Oxide Synthase 3 | CTGCATGACATTGAGAGCAAAG | CGGTAGAGATGGTCGAGCTG |
| IL10 | Interleukin 10 | TACAACAGGGGCTTGCTCTT | GCCAGGAAGATCAGGCAATA |
| IL6 | Interleukin 6 | CCTCTCCGGACAAAACTGAA | TCTGCCAGTACCTCCTTGCT |
| IL1B | Interleukin 1, Beta | TCTCTCACCCCTTCTCCTCA | GACCCTAGTGTGCCATGGTT |
| NOS2 | Nitric Oxide Synthase 2, Inducible | GCAGCTACTGGGTCAAGGAC | GCTGTTGGTGAACTTCCACTT |
| VCAM1 | Vascular Cell Adhesion Molecule 1 | CTTGACGTGAAAGGAAGAGAAAG | GGATGCACAATAGAGCACGA |
| OCLN | Occludin | GACGAGCTGGAGGAAGACTG | GTACTCCTGCAGGCCACTGT |
| CLDN1 | Claudin 1 | GGTCAGGCTCTCTTCACTGG | ATGTTGTTTTTCGGGGACAG |
| CASP3 | Caspase 3 | CTGGCAAACCCAAACTTTTC | GTCCCACTGTCCGTCTCAAT |
| TJP1 | Tight Junction Protein 1 | ATGACTCCTGACGGTTGGTC | TGCCAGGTTTTAGGATCACC |
| VEGFR2 | Vascular Endothelial Growth Factor Receptor 2 | ATCCCAGATGACAGCCAGAC | ATGGCGCTAATTTGGTTCTG |
| CLDN5 | Claudin 5 | CTGGTTCGCCAACATCGT | CAGCTCGTACTTCTGCGACA |
| HIF1A | Hypoxia Inducible Factor 1 Subunit Alpha | TGTGTTATCTGTCGCTTTGAGTC | TTTCGCTTTCTCTGAGCATTC |
| NFKB1 | Nuclear Factor Kappa B Subunit 1 | CCCTGTGAAGACCACCTCTC | ATCCCGGAGCTCGTCTATTT |
| VEGFA | Vascular Endothelial Growth Factor A | CGAAGGTCTGGAGTGTGTGC | TCTCTCCTATGTGCTGGCCT |
| BMP7 | Bone Morphogenetic Protein 7 | GACGCTGGTCCACTTCATC | GAAATGGCGTTGAGCTGAG |
| BMP2 | Bone Morphogenetic Protein 2 | AGACGTTGGTCAACTCCGTTA | CTCGTCAAGGTACAGCATGG |
| CDH1 | Cadherin 1 | ATGTGCACGTATGCGACTGT | GGAACTTGCAATCCTGCTTC |
| SOD2 | Superoxide Dismutase 2 | GATTGCCGCTTGTTCTAACC | TAATACGCATGCTCCCACAC |
| CASP8 | Caspase 8 | GCCCCCATCTATGATCTGAC | TATCCCCTTGACAAGCCTGA |
| ICAM2 | Intercellular Adhesion Molecule 2 | CGGACACCTCATTCACAGAG | TGCCACAAACAAGAAGAGCA |
| LCN2 | Lipocalin 2 | CAGTTCCAGGGGAAGTGGTA | GAGCTCGTAGGTGGTGGTGT |
| CXCL8 | C-X-C Motif Chemokine Ligand 8 | CTTCGATGCCAGTGCATAAA | CAGTGGGGTCCACTCTCAAT |
| HIF1AN | Hypoxia Inducible Factor 1 Subunit Alpha Inhibitor | GGCCCTGGTGATGTTCTTTA | GGCCCCCTTATACCAGAAGT |
| SOD1 | Superoxide Dismutase 1 | GATTCTGTGATCGCCCTCTC | CTGCCCAAGTCATCTGGTTT |
| GDNF | Glial Cell Derived Neurotrophic Factor | CGAAACCAAGGAGGAACTCA | CCGTCTGTTTTTGGACAGGT |
| RET | Ret Proto-Oncogene | TGCAGTACCTGGCTGAGATG | GGGACAGACCGAAGTCTGAG |
| SIX2 | SIX Homeobox 2 | GCGAGAACTCCAACTCCAAC | AGCTGCCTAGCACCGACTT |
| SIX1 | SIX Homeobox 1 | GTTCAAGAACCGAAGGCAAC | TGCTTGTTGGAGGAGGAGTT |
| WNT4 | Wnt Family Member 4 | AGGCTATCCTGACACACATGC | ACGTCTTTACCTCGCAGGAG |
| LRG1 | Leucine Rich Alpha-2-Glycoprotein 1 | AGGGCAATAGATTGCAGGTG | CGTGGTCAGCTTGTTGTTGT |
| REN | Renin | TCCACTACGTGAGCATCAGC | CCTCCTCACAGAGCAAGGTG |
| ACE | Angiotensin I Converting Enzyme | TCTGGAACAAGTCGATGCTG | AAGTCCTTGCCGTTGAAGAA |
| HCAR1 | Hydroxycarboxylic Acid Receptor 1 | GTGTTCATCACGGGCTACCT | CTACATGCACAGAGGGGTCA |
| SUCNR1 | Succinate Receptor 1 | CGTCAGCCTCACTTACAGCA | GGAAAAGGGCAATCTTGAAA |
| WNT11 | Wnt Family Member 11 | GAGCTCCCCTGACTTCTGC | GCTGTCACTGCCTTGTGATG |

**Supplemental Figure S1 Effects of asphyxia on blood gas parameters in vehicle-fed pigs from 5 litters during the first 24 h.** (A-F) The blood pH, lactate, pO_2_, pCO_2_, tHb, COHb, sO_2_ and base excess levels. All data for the control group (CON, n = 35) and asphyxia group (ASP, n = 27) are presented as the means ± SD.


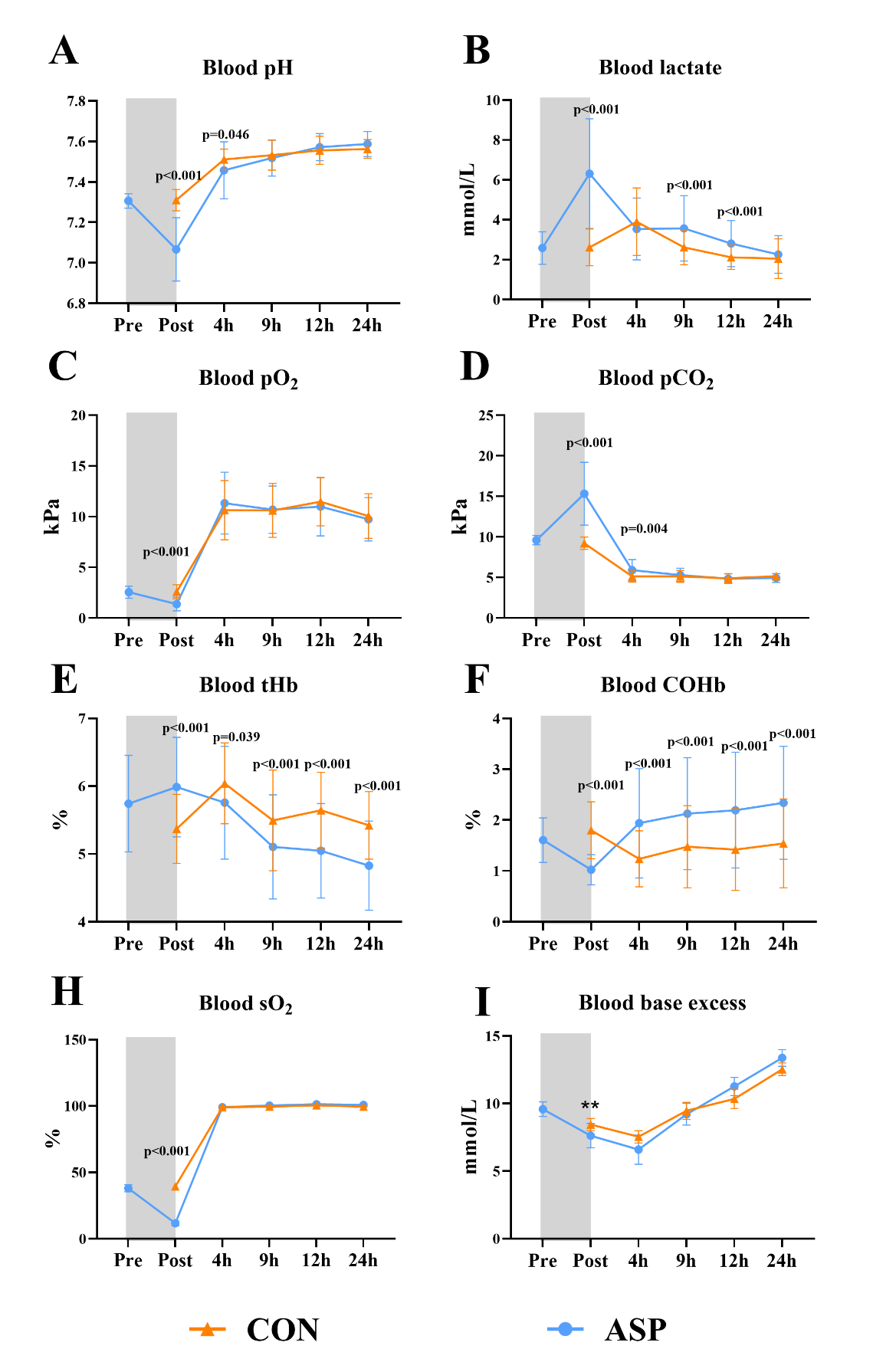


**Supplemental Figure S2** **Effects of asphyxia and plasma feeding on blood urea nitrogen to plasma creatinine ratio and plasma electrolytes in** pigs from the 72-h hour study**.** (A) The level of **blood urea nitrogen to plasma creatinine ratio**. (B-F) The levels of plasma Pi, K^+^, Mg^2+^, Ca^2+^ and Fe^2^. The n number for each group were as follows: CON-VEH (n = 20–27), CON-PLA (n = 20–25), ASP-VEH (n = 14–20), and ASP-PLA (n = 12–19). *P*_PLA_ indicates the significant impact of plasma feeding across all animals.


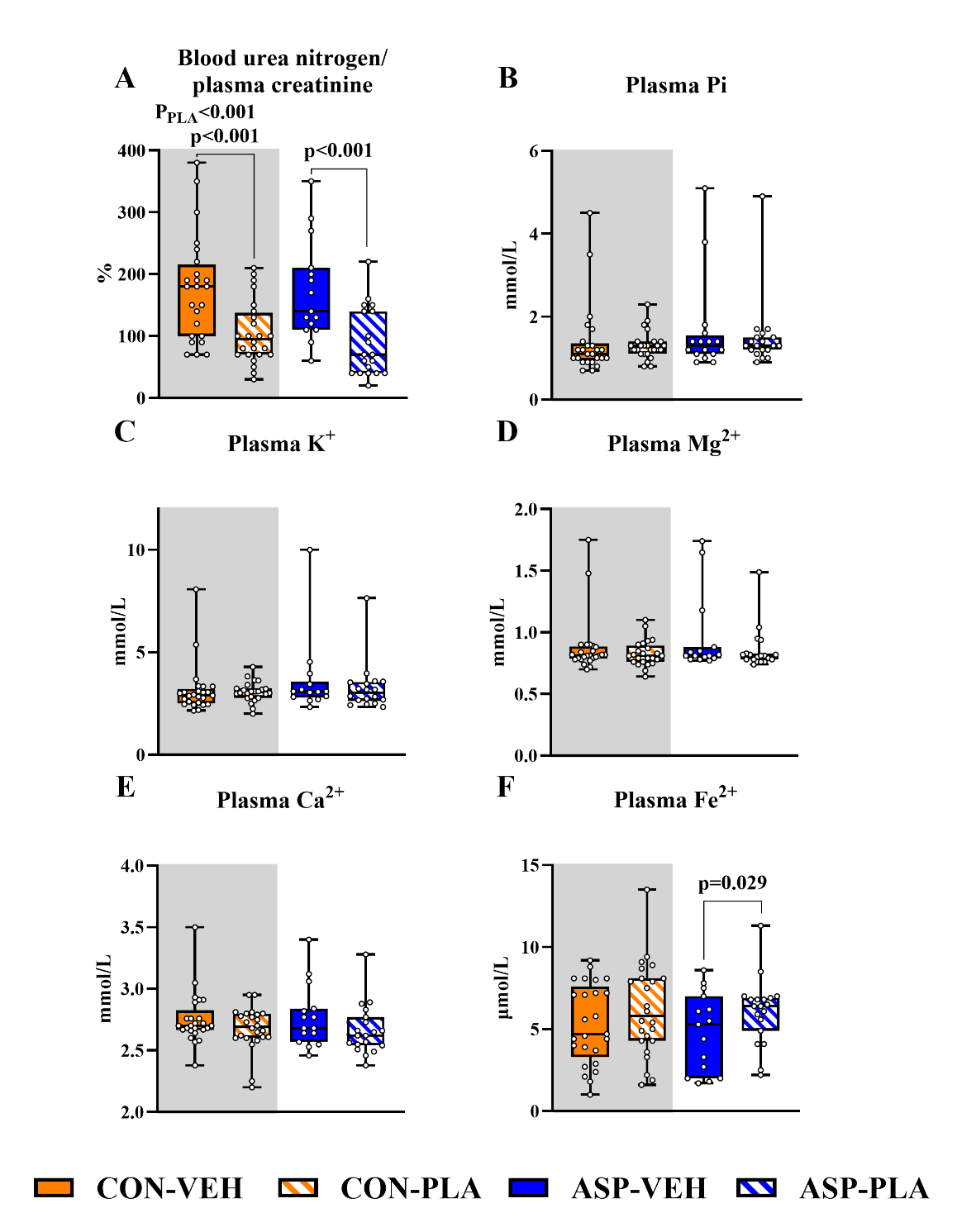


**Supplemental Figure S3** **Effects of asphyxia and plasma feeding on the prevalence of kidney lesions in** pigs from the 72-h study (evaluated by assessor 1)**.** (A-D) The prevalence of tubular vacuolisation, tubular dilatation, tubular necrosis, and focal interstitial inflammation, respectively. The sample size (n) for each group was as follows: CON-VEH (n = 28), CON-PLA (n = 24), ASP-VEH (n = 19), and ASP-PLA (n = 21).


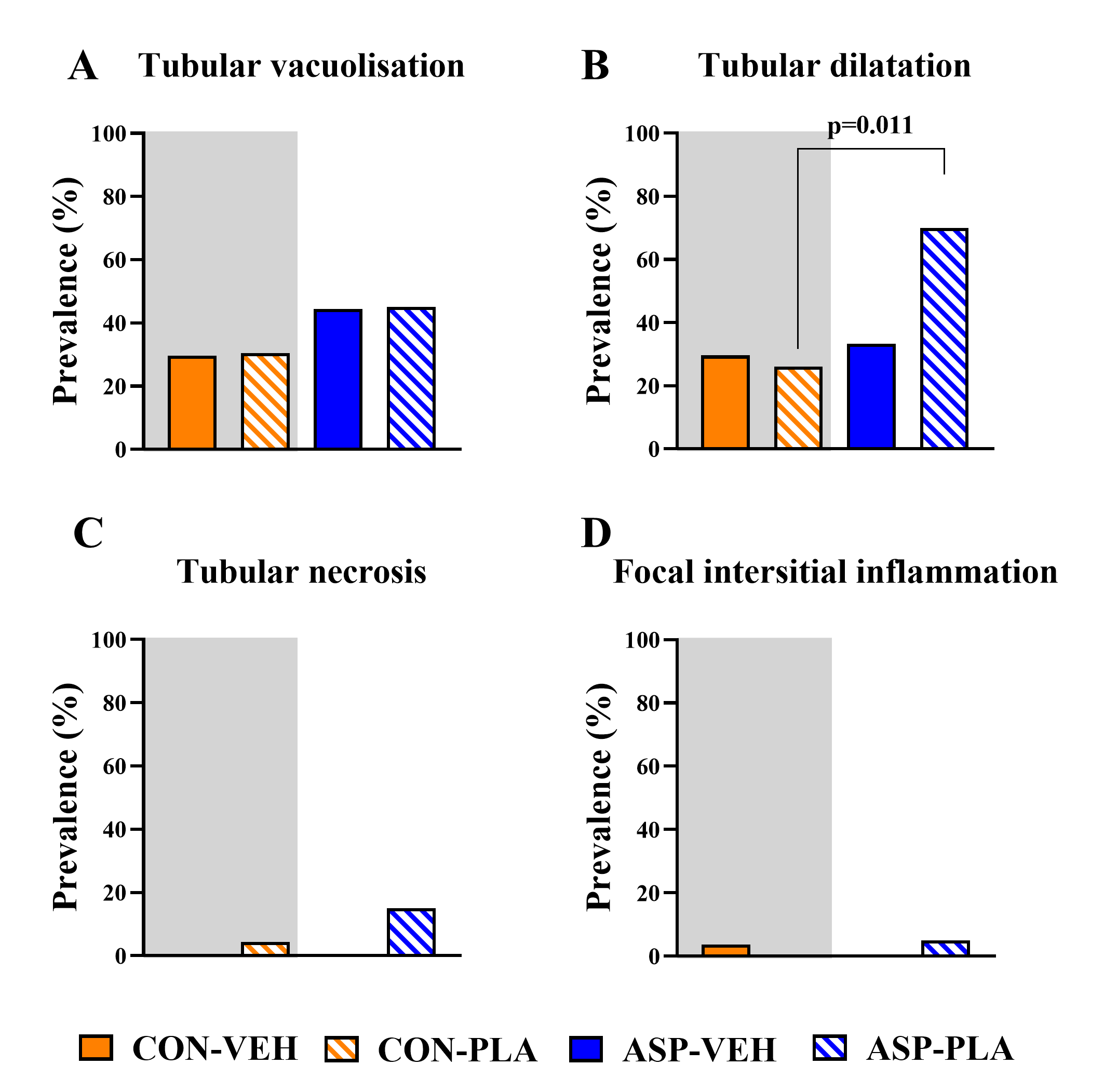


**Supplemental Figure S4** **Effects of asphyxia and plasma feeding on the prevalence of kidney pathologies in** pigs from the 72-h study (evaluated by assessor 2)**.** (A-H) The prevalence of tubular vacuolisation, tubular dilatation, lymphatic vessel dilatation, glomerular necrosis, glomerular hemorrhage, cystic dilation of Bowman's capsule, paracortical edema, and interstitial hemorrhage, respectively. The sample size (n) for each group was as follows: CON-VEH (n = 28), CON-PLA (n = 24), ASP-VEH (n = 19), and ASP-PLA (n = 21).


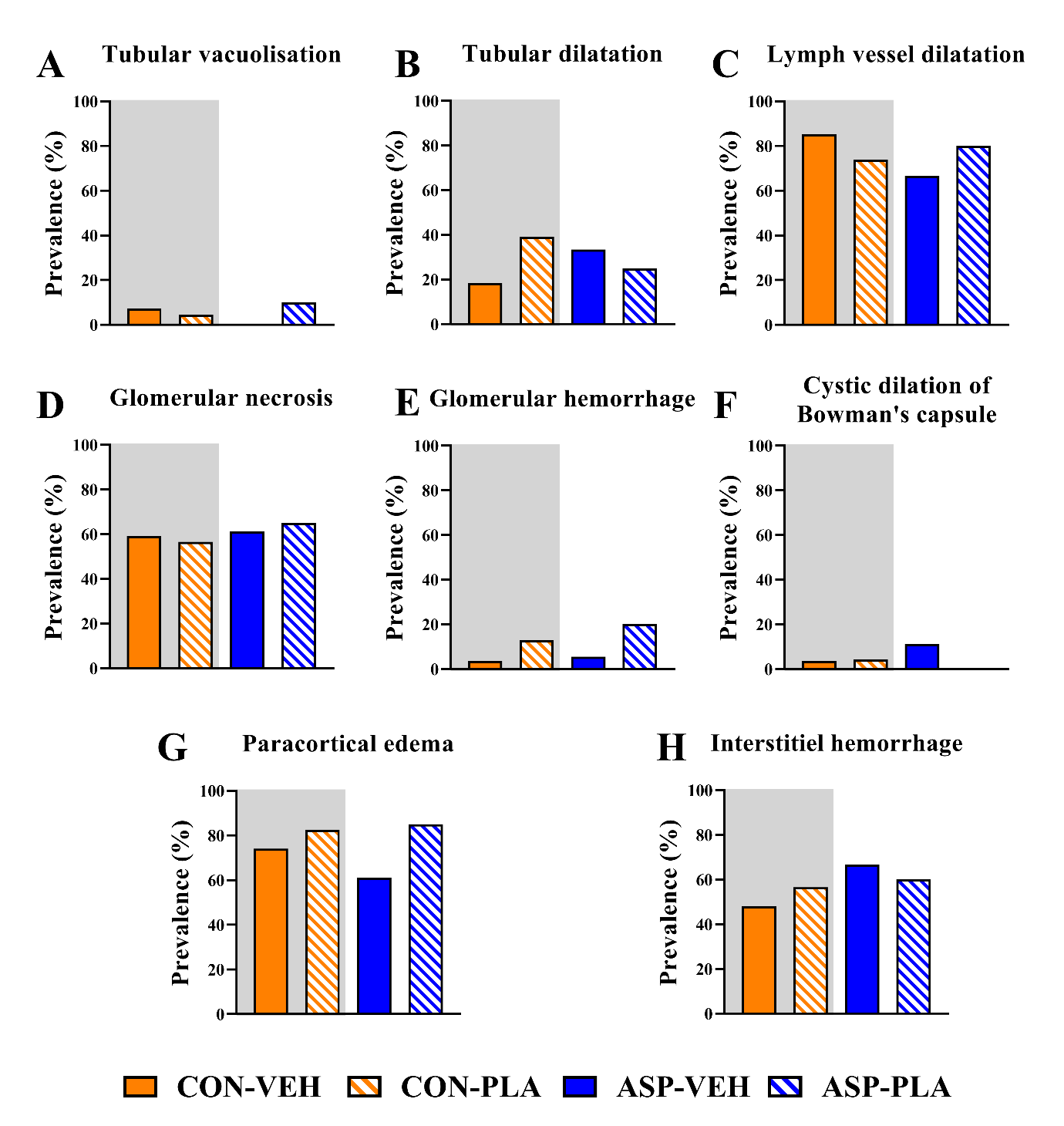

Supplement: Supplementary file 1 — Appendix S1. [file PHY2-13-e70238-s001.docx]
